# Upcycling end-of-life vehicle waste plastic into flash graphene

**DOI:** 10.1038/s44172-022-00006-7

**Published:** 2022-05-26

**Authors:** Kevin M. Wyss, Robert D. De Kleine, Rachel L. Couvreur, Alper Kiziltas, Deborah F. Mielewski, James M. Tour

**Affiliations:** 1 Department of Chemistry, Rice University, 6100 Main Street MS 222, Houston, TX 77005 United States; 2 Research and Innovation Center, Ford Motor Company, 2101 Village Rd., Dearborn, MI 48124 United States; 3 Smalley-Curl Institute, NanoCarbon Center, Welch Institute for Advanced Materials, Department of Materials Science and Nanoengineering, Department of Computer Science, Rice University, 6100 Main Street MS 222, Houston, TX 77005 United States

**Keywords:** Composites, Chemical engineering

## Abstract

Responsible disposal of vehicles at the end of life is a pressing environmental concern. In particular, waste plastic forms the largest proportion of non-recycled waste material from light-duty vehicles, and often ends up in a landfill. Here we report the upcycling of depolluted, dismantled and shredded end-of-life waste plastic into flash graphene using flash Joule heating. The synthetic process requires no separation or sorting of plastics and uses no solvents or water. We demonstrate the practical value of the graphene as a re-inforcing agent in automotive polyurethane foam composite, where its introduction leads to improved tensile strength and low frequency noise absorption properties. We demonstrate process continuity by upcycling the resulting foam composite back into equal-quality flash graphene. A prospective cradle-to-gate life cycle assessment suggests that our method may afford lower cumulative energy demand and water use, and a decrease in global warming potential compared to traditional graphene synthesis methods.

## Abstract (web summary)

Kevin Wyss and colleagues report the flash synthesis of graphene from end-of-life vehicle plastic waste. A polyurethane/flash graphene composite is also re-flashed back into more graphene. A life cycle assessment suggests environmental benefits compared to other graphene synthetic routes.

## Introduction

Due to a decreasing cost of entry and an increasing global standard of living, automobile access has expanded ownership to record highs, with an estimated 1.4 billion passenger cars in use worldwide^1,2^. Inevitably, these vehicles come to the end of their useful life and must be managed^3,4^. End-of-life vehicles (ELV) present a complex environmental problem due to their heterogenous construction and ever-advancing robustness^5^. Processing standards vary broadly worldwide, but in the US as of 2020, depollution (removal of fluids and batteries) and dismantling remove 10–30% of the raw vehicle weight while the remainder is shredded^6,7^. These percentages vary by vehicle identity and construction. The metallic content is largely recovered, but the remainder of the ELV (12 to 32% of the raw vehicle weight) is typically landfilled^8–10^. The amount of plastic used in vehicles has increased an estimated 75% in the past 6 years to 350 kg per vehicle for weight reduction to improve fuel economy^11^. ELV waste plastic (ELV-WP) is the largest non-recycled material in vehicles, and the increased use of next-generation polymer composites exacerbates recycling of ELV-WP through traditional methods which generally focus on singular plastic sources^12,13^. Recycling or upcycling of ELV-WP is economically unfavorable due to the high cost of feedstock segregation^12,13^. Some nations have set mandatory recycling/recovery goals in attempts to minimize environmental impact while maximizing resource reclamation. For instance, the European Union implemented the ELV Directive to ensure that recovery of ELV raw materials achieves a minimum of 95% of vehicle weight by 2015; however, almost all member states failed to meet these guidelines^14^. Even with governmental policy incentives, ELV-WP management remains a dilemma. As ELV-WP draws more global attention, a number of strategies have been proposed by academic as well as automotive sectors. More wholistic understanding of the problem has recently been made possible by bottom-up analysis of the ELV waste management industry and life cycle assessments on a region by region basis, with many studies and reviews being recently published^15–17^. Generally, the ELV waste management strategy depends on the socioeconomic status of the region or country, as well as the systematic techniques required by the governing body^18^. Polypropylene often receives the most academic interest, as it is the most used plastic in automotive applications; many bumpers are made of polypropylene making for easier isolation of a single type of plastic for recycling. Recent lab-scale remediation strategies revolve around pyrolysis methods; however, these often require complex catalysts, inert atmospheres, and they struggle to recycle dirty or mixed streams of ELV-WP^19,20^.

The automotive sector produces an estimated 5% of the global industrial waste in the form of ELV, however given low virgin polymer costs there is little attraction to pursue ELV-WP recycling. While the automotive sector follows regional or government directives, it has been slower to confront the plastic waste problem^21^. Unlike valuable metal or electronic automotive components, there is little economic incentive to recover and recycle ELV-WP^22^. Many automotive manufacturers are studying novel ‘green’ polymers or composites and the use of more sustainable polymer reinforcements such as cellulose, waste textiles, agave fibers, or polymer waste^23–26^. These goals and products may markedly decrease the burden of producing virgin materials for new automobiles, but they do not combat the 1.4 billion passenger vehicles that contain >10^12^ kg of ELV-WP. For ELV-WP remediation to prosper outside of highly variable regional regulations, a high value upcycled or recycled product should be accompanied with minimal separation requirements, low-cost infrastructure, and a low energy and material overhead.

Recently, a process to convert mixed waste plastics into graphene was reported^27,28^. This method took advantage of flash Joule heating (FJH), a highly efficient technique using small amounts of electrical energy to form high quality turbostratic graphene called flash graphene (FG). This is a solvent-, water-, and furnace-free method at a projected electrical energy cost of only ~$125 per ton of plastic waste^27^. Graphene is an extremely valuable material (retail prices $60,000 to $200,000 per ton) due to its useful properties^29^. For example, graphene has a Young’s modulus of 1 TPa, with good electrical conductivity and high thermal and chemical stabilities^30^. Thus, graphene has received considerable attention as a composite additive to enhance the properties of the host material, in applications ranging from plastics to concrete and asphalt^16–18^. This allows for less host material to be used to achieve the same properties, decreasing the overall environmental footprint of the host.

The FJH process for producing FG is currently being rapidly scaled to multi-ton per day production, suggesting the feasibility of applying this process to ELV-WP^31^. The objectives of this paper include demonstrating the FJH upcycling of ELV-WP into FG, which is then used to enhance vehicle polyurethane foam (PUF) composites. Another objective is to conduct a prospective life cycle assessment (LCA) comparing FJH to other graphene production methods, and to demonstrate the continuous upcycling of FG-enhanced composites.

## Results

### Flash graphene preparation

FJH uses a fast discharge process through a resistor with little energy passing through the surrounding system^15,16^. Here, the resistor is the ELV-WP contained in a quartz tube, and the heat is generated directly within the plastic feedstock, eliminating slow and inefficient heat transfer that traditional tube furnaces experience. The ELV-WP is converted as a mixture, and there are no known plastic compositional limitations for the process. The ELV-WP used here is an output of the shredding process, after depollution and dismantling steps. The large plastic chunks yielded by the automobile shredder were ground to a 1 mm particle size and used without any further purification. The yield of ELV-WP-FG is dependent on the amount of carbon present in the plastic. For example, HDPE contains 86% carbon by mass, whereas poly(vinyl chloride) contains 38% carbon by mass.

**Fig. 1:**
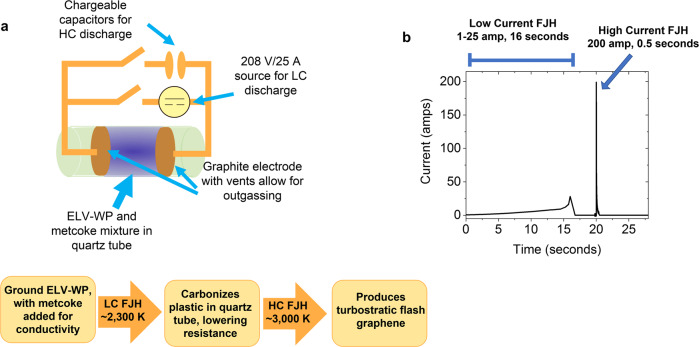
Process schematic, workflow, and current discharge for FJH conversion of ELV-WP into FG. **a** Block diagram of the custom designed dual capability FJH station for low current (LC) and high current (HC) discharge, with a working procedure displayed below. **b** A typical current discharge profile of the FJH procedure to convert ELV-WP into ELV-WP-FG.

Over the course of the current discharge through the ELV-WP resistor, extremely high temperatures (2300 to 3000 K) are achieved in seconds^32–35^. The experimental setup and current profile are shown in Fig. 1. The current discharge is separated into two distinct regions: a low current (LC) region and a high current (HC) region (Fig. 1b). During the LC discharge, lasting 10 to 16 s, current of 1–25 A is applied at a constant voltage of 208 V, with the current increasing as the plastic is carbonized^27^. Initially the plastic mixture, 95% finely ground plastic and 5% finely ground metallurgical coke (metcoke, a low-cost commercial carbon product derived from coal) for conductivity enhancement, has a resistance of ~500 Ω, limiting the current. However, heat generated during LC-FJH carbonizes the plastic, increasing the conductivity and further increasing the applied current until a resistance of ~10 Ω is reached. Temperatures of ~2300 K are achieved by the end of the LC-FJH process, measured by an IR thermometer^27^. Approximately 30% of the initial ELV-WP is recovered as highly carbonized plastic, with volatile outgassing and sublimation of contaminants resulting in the loss of weight. The evolved gases have been previously studied, showing that an estimated approximate pressure ratio of 5:4:1 H_2_/C_1–3_/C_4–6_ is observed for high-density polyethylene^27^. Similarly, hydrocarbon-rich waxes and oils can also be recovered after the LC-FJH process^27^. Next, the HC-FJH process is used, applying ~200 A at an initial voltage of 150 V through the carbonized sample in <1 s (Fig. 1b), efficiently converting the carbonized plastic into FG through the breaking and reordering of C-C bonds into the thermodynamically favored sp^2^-hybridization of graphene^28^. Hydrogen, oxygen, chlorine, silica, and trace metal impurities sublime and outgas during the FJH process due to the high temperatures achieved^33^. The HC-FJH process yields 85% mass recovery from the carbonized ELV-WP for a theoretical total recovery of ~25% mass from the raw ELV-WP starting material. Further process details are provided in the “Methods” section.

Bumpers, gaskets, carpets, mats, seating, and door casings derived from ELV Ford Motor Company F-150 pickup trucks were milled together to demonstrate the general process applicability. The ELV-WP was ground using a hammer mill with no washing, separations, or sorting, combining poly(vinyl chloride), acrylonitrile butadiene styrene, polypropylene, polycarbonate, polyamides, polyoxymethylene, polyurethane and polyethylene. To increase the conductivity of the mixture for the FJH reaction 5 wt% ground metcoke was added. For this study, a singular LC/HC FJH station was built which could provide both the LC and HC treatments, respectively. Extensive discussion on LC/HC FJH station design and experimental setup are in Supplementary Fig. 1. With the LC/HC system, synthesis of the FG from ELV-WP was straightforward. Following FJH, yields of 19 to 24% were observed on 20 batches, yielding 11 g of ELV-WP-FG.

### Flash graphene characterization

**Fig. 2:**
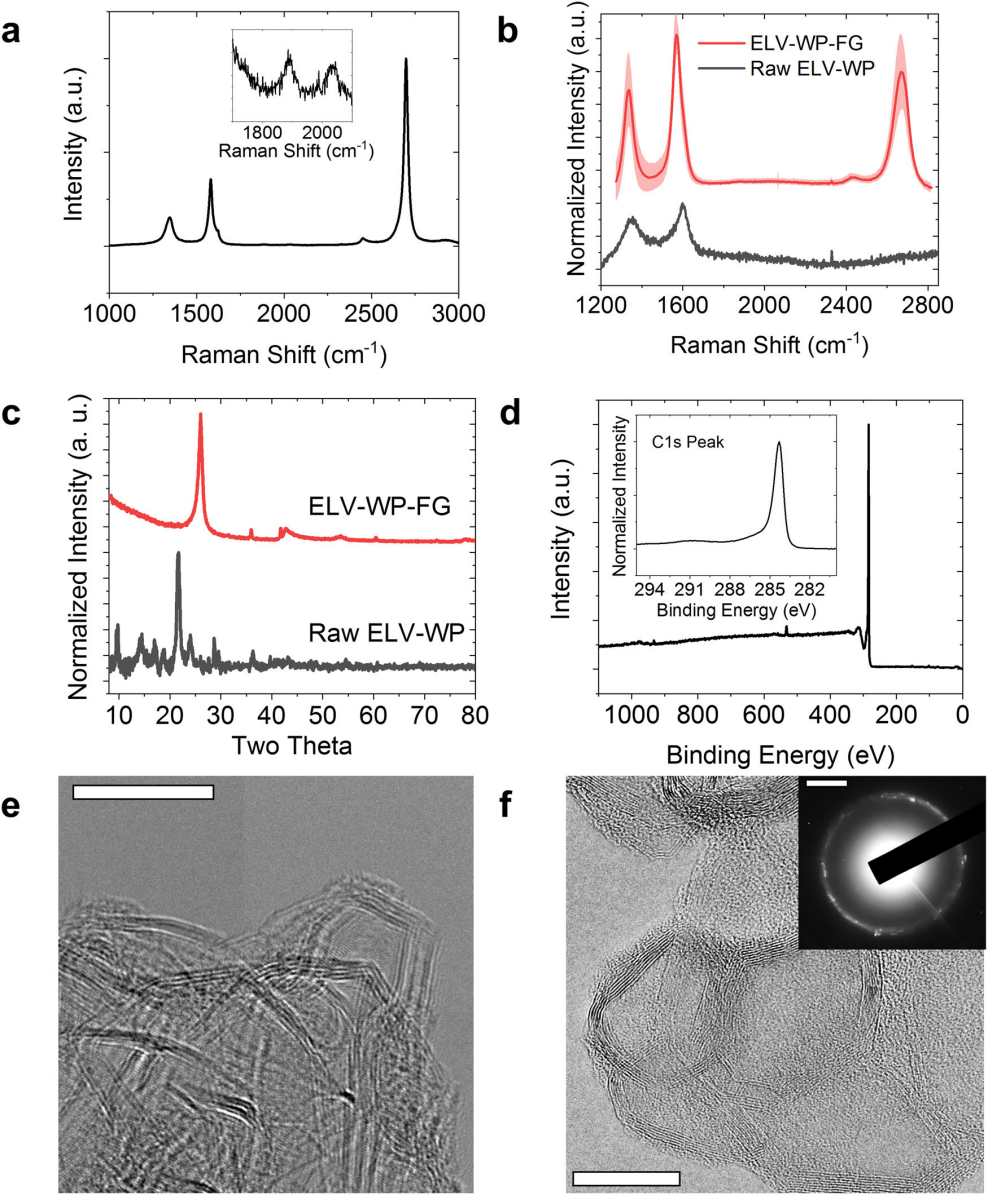
Characterization of produced ELV-WP-FG. **a** A sample Raman spectrum, with expanded inset showing turbostratic indicators. **b** An average Raman spectrum derived from 225 individual spectra, with the shaded area representing the standard deviation, as compared to a typical spectrum of the starting ELV-WP. **c** Powder X-Ray diffraction comparing ELV-WP-FG to ELV-WP. **d** XPS survey scan of ELV-WP-FG, with inset high-resolution spectra of the C1s transition. **e** TEM image of ELV-WP-FG, scale bar is 10 nm. **f** TEM image of ELV-WP-FG (scale bar is 10 nm), with inset selected area electron diffraction (scale bar is 5 nm^−1^) demonstrating rotational disorder or turbostraticity.

Several analytical methods were used to confirm the high quality of the produced FG. Raman spectroscopy probes the composition, structure, and arrangement of the 2D graphene sheets^36–38^. A typical Raman spectrum (Fig. 2a) of the ELV-WP-FG shows that the FG is high quality due to the intense and narrow 2D peak at 2690 cm^−1^, indicative of the long-range hexagonal bonding. The noticeably less intense D peak at 1350 cm^−1^ indicates that there are few lattice defects, such as breaks in symmetry, holes, or edges^39^. Further, the ability to fit a single Lorentzian function to the 2D peak indicates that the FG is optically decoupled from the neighboring layers, characteristic of turbostratic arrangement^40^. A high 2D/G peak intensity ratio is indicative of high quality graphene with little interlayer optical coupling^40^. This decoupling between layers is presumably due to the rapid cooling rate, kinetically trapping the FG sheets in a state of rotational disorder since they do not have time to stack into an AB (Bernal) form. This rotational disorder in turbostratic FG lessens many of the Van der Waals interactions when compared to AB-stacked graphene^40^. These interlayer coupling interactions in AB-stacked graphene are less preferred since they make exfoliation difficult, while exfoliation of FG is facile. The presence of the TS_1_ and TS_2_ peaks at 1875 cm^−1^ and 2025 cm^−1^ in the Raman spectrum, respectively, confirm the rotationally disordered stacking (Fig. 2a inset)^41^. The absence of the M peak, normally at 1750 cm^−1^ and indicative of AB repetitive stacking in graphite, again shows that the product is turbostratic^41^.

To better characterize the quality and homogeneity of the many batches of ELV-WP-FG, they were mixed together using a mortar and pestle, and 225 individual Raman spectra were collected. The average spectrum with standard deviation is shown in Fig. 2b, demonstrating good bulk homogeneity and quality is achieved. Of the 225 spectra, 94.6% were determined to be graphene (using 2D/G > 0.3 as the standard), indicating that very high degrees of bulk conversion are attained. An average 2D/G ratio of 0.81 and average D/G ratio of 0.58 are observed, with an average 2D peak full width at half maximum (FWHM) of 54 cm^−1^.

Powder X-ray diffraction (XRD) further probes the bulk homogeneity of the FG. As shown in Fig. 2c, the (002) and (100) peaks corresponding to FG are present at 26.1° and 43.3°, respectively. These peaks are distinct from graphite because of the shift toward lower diffraction angles (26.1° for FG vs. 26.6° for graphite) and wider FWHM^28^. No other intense peaks are present, confirming the successful conversion of the polymer precursor and removal of the crystalline contaminants from the starting composite fillers. The purity of the ELV-WP-FG was further assessed with X-Ray photoelectron spectroscopy (XPS). Survey scans show that the sample is 98% carbon, with the remaining 2% oxygen and no noticeable impurities, to the limits of bulk XPS detection. A high-resolution spectrum of the C1s peak (Fig. 2d) shows that the carbon bonding character is solely sp^2^/sp^3^, with no discernable oxygen functionalization. Further, the π-π* transition is apparent at 291 eV.

The graphene sheets, with average sheet size of 13.8 ± 7.1 nm, can be visualized using transmission electron microscopy (TEM) (Fig. 2e, f). The small sheet size and large size distribution is due to the bottom-up synthetic method of FJH. Previous reports have demonstrated that the sheet size and morphology of FG is kinetically controlled and feedstock dependent^27,28,34,42,43^. The observed interlayer spacing is increased to 0.358 nm for FG vs. 0.334 nm for graphite. The rotational disorder causing this increased spacing can be observed from the selected area electron diffraction pattern. Further characterization of the ELV-WP-FG was done to study the thermal stability using thermogravimetric analysis on the weight loss (Supplementary Fig. 2). Under air atmosphere, a single degradation is observed at 500–650 °C, showing much higher stability than the starting ELV-WP. The surface area of the ELV-WP-FG was also studied using Brunauer-Emmett-Teller (BET) gas adsorption. The BET analysis demonstrated a specific surface area of 60 m^2^ g^−1^ and a cumulative pore volume of 0.23 cm^3^ g^−1^ as well as a mesoporous pore size distribution (Supplementary Fig. 3). Although this specific surface area is somewhat lower than physically exfoliated graphene powders, the dispersibility is far better than commercial graphene due to the turbostratic stacking. Also, the low surface area did not negatively impact the composite properties of the material as demonstrated in PUF in the “Application of flash graphene in polyurethane foams” section.

**Fig. 3:**
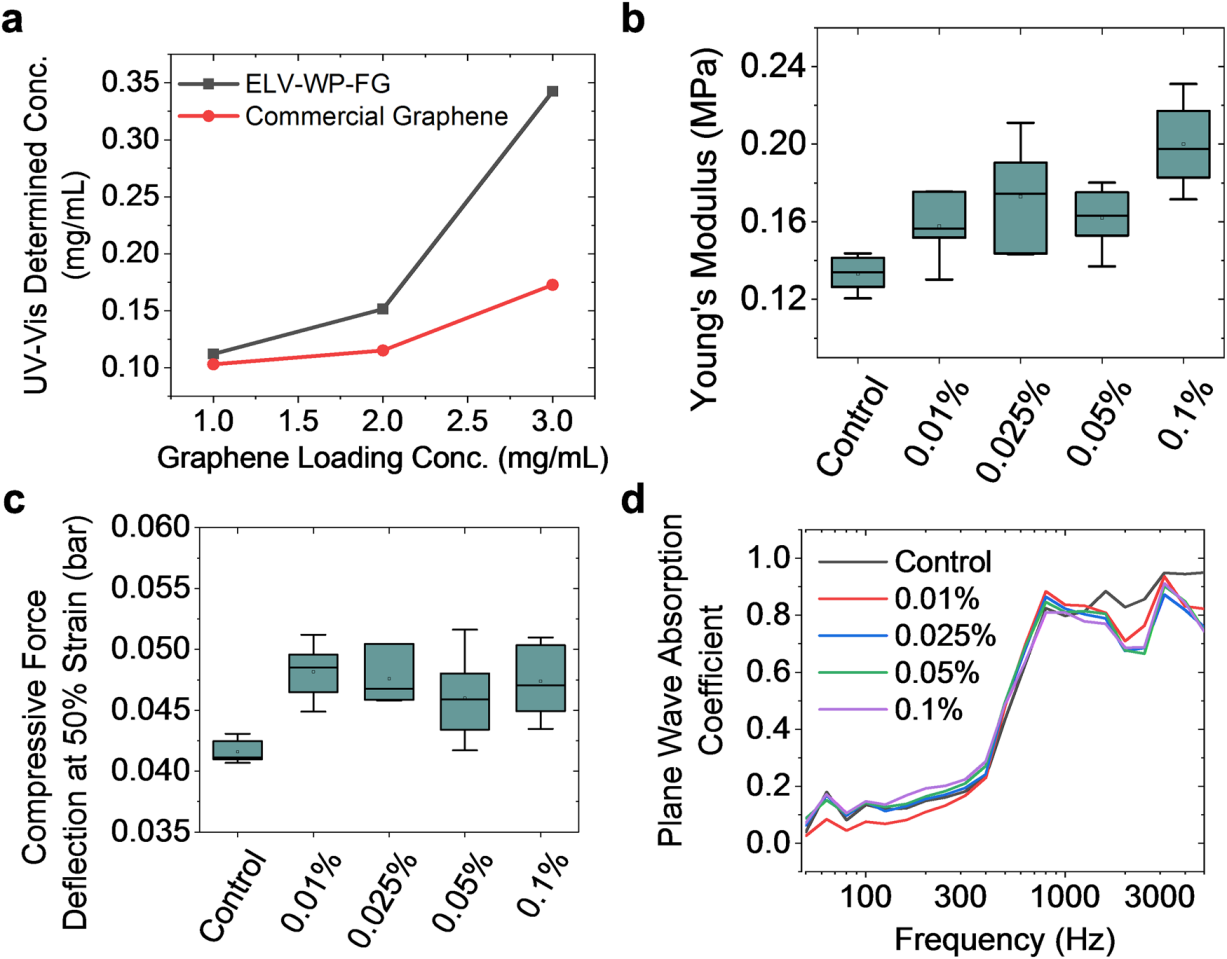
Dispersibility of FG and mechanical properties of FG-enhanced PUF composites. **a** Dispersion of ELV-WP-FG compared to commercially available graphene in 1% Pluronic-F127 non-ionic surfactant assisted aqueous system. The mechanical properties of ELV-WP-FG/PUF composites including **b**, the Young’s Modulus (*N* = 6), **c** compressive force deflection at 50% strain (*N* = 8), and **d** plane wave noise absorption coefficient (*N* = 4, line represents average plot). The interquartile range is shown by shaded “box”, while the maximum and minimum are shown by the “whiskers”.

A characteristic of FG that is essential to successful implementation in composites is its exfoliation and dispersibility in media. As highlighted above, turbostratic stacking weakens the interactions between the FG layers allowing sonication to make stable dispersions of FG in a variety of solvents^27,28,32,33^. The amount of graphene dispersed in a solution is determined by UV-Vis adsorption. After the graphene is added and sonicated, centrifugation is used to remove larger non-dispersed aggregates. ELV-WP-FG demonstrates a dispersibility of 0.35 mg ml^−1^ at an initial loading concentration of 3 mg ml^−1^ as shown in Fig. 3a. Compared to commercially available graphene synthesized through physical exfoliation methods (Tianyuan Empire Materials, Shatin, Hong Kong), the ELV-WP-FG was twice as dispersible. The UV-Vis spectra for the highest concentration commercial graphene and ELV-WP-FG dispersions can be found in Supplementary Fig. 4.

### Application of flash graphene in polyurethane foams

With the bulk conversion and dispersibility of ELV-WP into FG demonstrated, use of ELV-WP-FG in PUF was then explored. PUF materials are used extensively in automobile components that require sound damping and vibration management, including engine covers, dashboard silencers, seat cushions, and more^44,45^. Since February 2020, Ford Motor Company has used graphene, obtained through graphite exfoliation, in all of its vehicles for PUF enhancement and weight reduction^46^. PUF is a carbamate-containing rigid polymer that is formed through the reaction of isocyanate and polyol resulting in an alternating copolymer. During the polymerization, a blowing agent is commonly used to create the open cell porous foam that results in a low-density foam. Addition of water during the polymerization step is most common industrially, which evolves CO_2_ gas upon reaction with the isocyanate, physically forming the pores. In this work, the ELV-WP-FG powder was added to the solution of polyol with water, catalyst, and surfactant added to tailor the properties of the final PUF. The graphene was dispersed through brief shear mixing, followed by the addition and mixing of the isocyanate. The reaction mixture is then added to a mold and heated to speed the reaction. The final PUF is then cut into cubes for mechanical testing and physical characterization. Further fabrication details can be found in the “Methods” section.

### Characterization of polyurethane foam

Over a series of mechanical, thermal and processability studies, ELV-WP-FG as prepared here was shown to be a similar enhancer to PUF. Optical images of the ELV-WP-FG-PUF cubes can be seen in Supplementary Fig. 5. Upon addition of 0.01 to 0.1% of ELV-WP-FG to PUF, the density of the composite changed little (Supplementary Fig. 6). However, these loadings of ELV-WP-FG increased the Young’s Modulus (Fig. 3b), by a maximum 34% at 0.1% FG. The increase did not plateau vs. loading, so tensile strength might increase even more at higher loadings. Further, it was observed that the compressive force deflection at 50% strain was increased by 19%, at as low as 0.01% ELV-WP-FG loading (Fig. 3c). The compressive force deflection is an important property of foam materials since it reflects the resistance to force applied to the surface and the corresponding amount of deformation that can be expected. The extension at maximum load, tensile strength at maximum load, compressive modulus, and tear resistance of the ELV-WP-FG-PUF composites were also studied with little change in the PUF properties (Supplementary Figs. 7–10). Graphene is also known for its sound absorption properties^47,48^. Thus, the acoustic absorption of the ELV-WP-FG-PUF composites were tested and showed increases in sound absorption at low frequencies from 50 to 300 Hz with a sharp increase from 300 to 3000 Hz (Fig. 3d). Up to 30% increase in absorption was observed at 200 Hz, and higher ELV-WP-FG loadings might further improve acoustic absorption. A comparison of the ELV-WP-FG-PUF at 0.01, 0.025, 0.05, and 0.1% loadings to the control PUF sample with no added graphene is presented in Table 1. Representative tensile and compression curves for each sample type are provide in Supplementary Fig. 11. The hydrophobicity of the ELV-WP-FG-PUF samples was compared to the hydrophobicity of control PUF samples with no added ELV-WP-FG. The control sample PUF exhibited a contact angle of 88.2° whereas the ELV-WP-FG-PUF sample with 0.1% added graphene demonstrated a contact angle of 101.6° (Supplementary Fig. 12). This contact angle can be compared to the contact angle of the FG film and PUF derived FG film formed by vacuum filtration of dispersions, which show contact angles of 137° and 126°, respectively (Supplementary Fig. 13).

**Table 1:** Comparing the physical properties of ELV-WP-FG-PUF 0.01, 0.025, 0.05, and 0.1% to the control PUF sample with no added grapheme.

	Control	0.01% FG	0.025% FG	0.05% FG	0.10% FG
Young’s modulus (MPa)	0.139	0.158	0.173	0.162	0.187
Compressive force deflection (bar)	0.0406	0.0482	0.0467	0.0460	0.0474
Extension at max load (mm)	96.961	84.461	82.933	73.905	87.239
Tensile strength at max load (Kpa)	81.517	73.650	75.933	58.000	89.100
Compressive modulus (bar)	0.2708	0.2674	0.2692	0.2741	0.2823
Tear resistance (N mm^−1^)	0.3180	0.3123	0.3226	0.2726	0.2608
Sound absorbance (200 Hz)	0.137	0.110	0.155	0.164	0.193
Density (kg m^−3^)	42.10	43.83	42.19	43.85	42.89

**Fig. 4:**
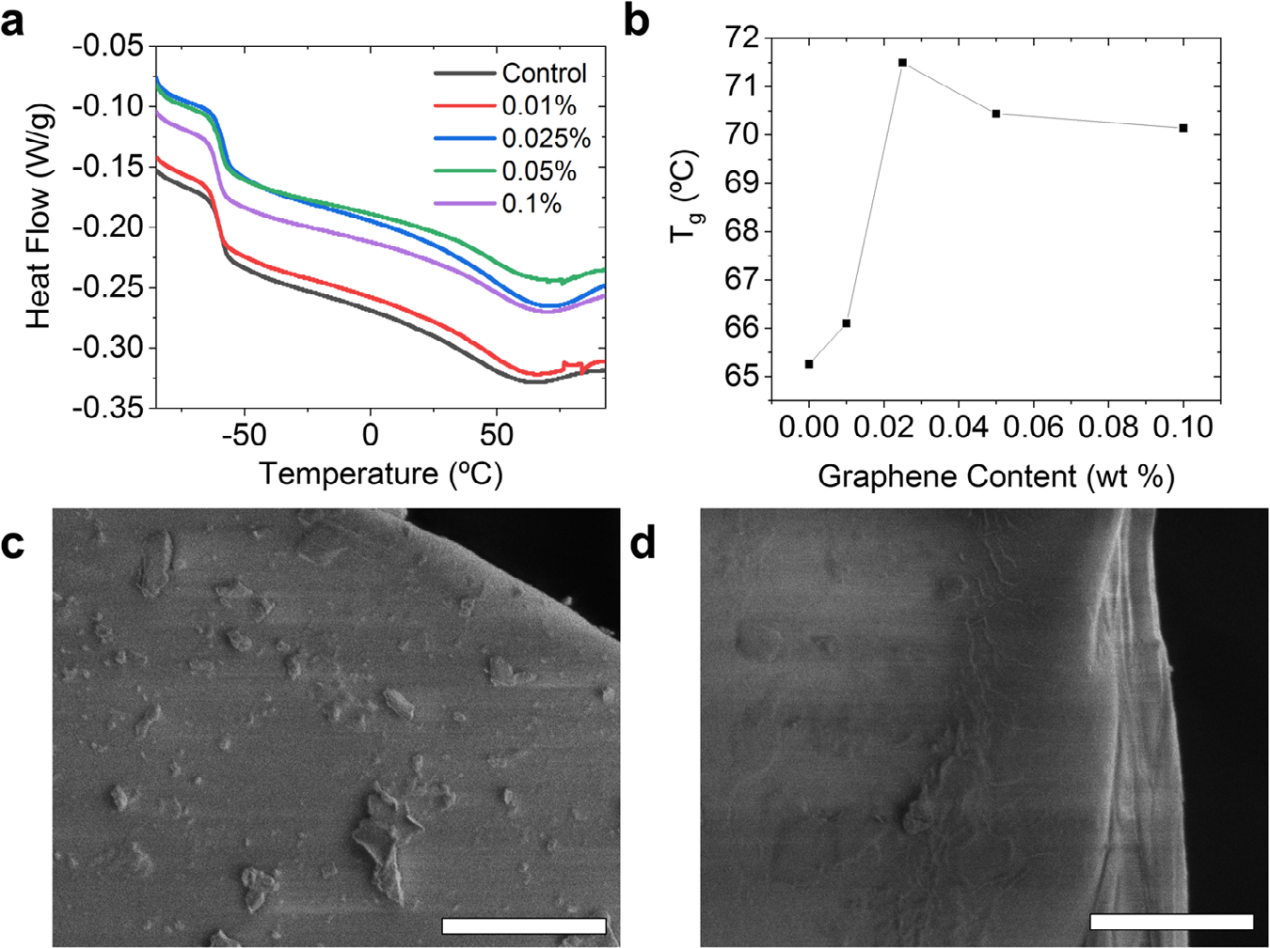
Physical properties and cross-sectional imaging of FG-enhanced PUF composites. **a** Differential scanning calorimetry of the ELV-WP-FG/PUF composites showing the beta transition and T_g_. **b** A plot of glass transition temperature as a function of graphene content, determined by the local minima of the derivative of the DSC heat flow. **c**, **d** Cross-sectional SEM analysis of ELV-WP-FG-PUF composite and neat PUF, respectively. The scale bars for both images are 5 μm.

Both good dispersion of the nanomaterial in a host matrix and good interfacial interaction between the host and the additive are essential for composite enhancement^49,50^. Study of the ELV-WP-FG-PUF composites interactions was done using differential scanning calorimetry (DSC) and cross-sectional scanning electron microscope (SEM) analysis. The results, shown in Fig. 4, demonstrate that the addition of even small amounts of FG can result in increases in the glass transition temperature (T_g_) of PUF from 65 to 72 °C. This increase results from interactions between the polymer chains with the FG sheets, impeding their reptation. A slight increase in the beta transition temperature, a relaxational transition similar to T_g_, can also be observed from −63 to −60 °C. The derivative of the heat flow affords a clearer representation of the increase in T_g_, as shown by a plot of the local minima temperature in the heat flow in Fig. 4b. The non-linear increase in T_g_, and largest increase being observed in the 0.025% FG sample, might point to high concentration aggregation of the ELV-WP-FG occurring during the polymerization or foaming steps. The cross-sectional SEM images confirm that ELV-WP-FG aggregation might be occurring during the foaming process, since larger particles are observed in 0.1% ELV-WP-FG-PUF. The surface morphology of the PUF can depend on the blowing agent used, along with the properties of the polyol used^51–53^.

### Comparative prospective life cycle assessment of flash graphene

Due to the solvent-free nature of the FJH synthetic method, and no requirement for lengthy furnace heating or inert gas atmosphere, it is hypothesized that the process offers noticeable environmental improvements. Quantification of resource consumption and environmental impact is an essential thrust in green chemistry^54^. Due to the lack of standardization surrounding the synthesis, categorization, and quality of commercial graphene, it remains a challenge to directly compare environmental and economic values^55^. However, a few LCAs have estimated the cumulative energy, emissions, and/or water requirements of the graphene synthesis processes^56–58^. We estimated these metrics for the FJH process in order to compare to competing processes.

### LCA goal and scope

A prospective LCA was conducted for FG produced from FJH and compared with graphene produced from graphite exfoliated by sonication or by oxidation to graphene oxide followed by chemical reduction. The study goal was to compare the cradle-to-gate impacts among these three alternative graphene synthesis pathways, and thus graphene use and disposal was excluded. A functional unit of 1 kg of graphene powder was considered. Powdered graphene rather than solution phase dispersed graphene was modeled since it is of wider utility industrially and lighter to ship. The scope of this LCA considers the cumulative energy demands (CED), global warming potential (GWP) over a 100-year timescale, and cumulative water use (CWU). The scope and life cycle inventory of this LCA does not include one-time impacts such as factory construction and land use, or manufacturing of the one-time components (i.e., reactor tanks) and machines (i.e., automotive shredder) necessary to produce the raw materials or carryout the process workflow. This scope also does not include plant burdens, such as HVAC, lighting, supporting activities for materials handling, quality control, or packaging.

### LCA methods

Process input and output data for sonication and chemical pathways were based on literature. Material transportation or waste stream disposal/remediation were defined as outside the scope of this limited study. Background data was principally sourced from Argonne National Laboratory’s GREET model including both GREET.Net software and spreadsheet models^59^. A detailed spreadsheet of process inputs and outputs as well as inventory and impact calculations can be found in Supplementary Tables 3–6. The LCA data are also uploaded as an editable spreadsheet in the Supplementary Data 1.

**Fig. 5:**
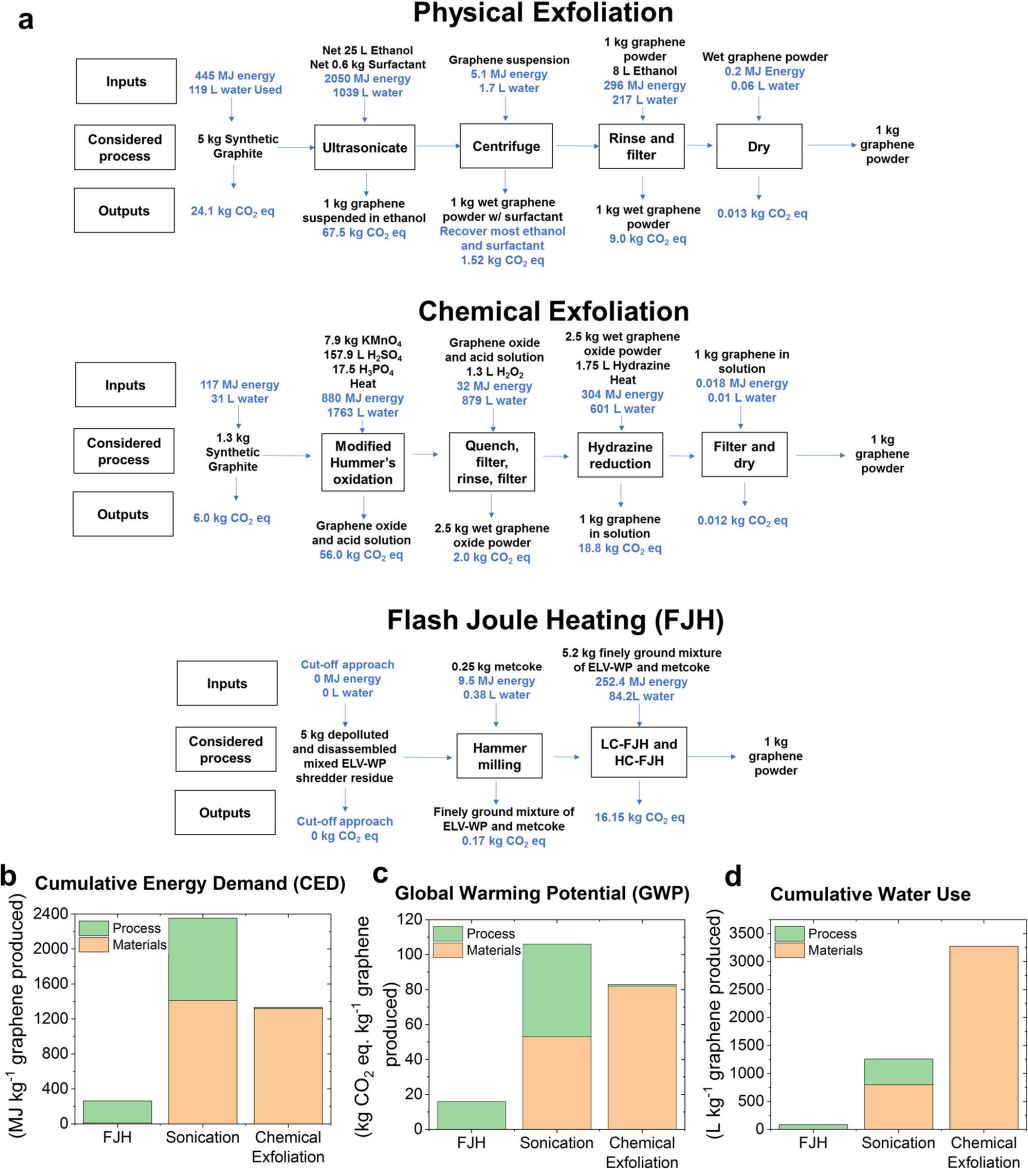
Process flow and life-cycle assessment comparing graphene synthesis methods. **a** Process flow diagrams of various graphene synthetic routes, displaying the lifecycle inventory including all considered inputs and outputs. Incidental inputs and outputs are shown in blue font to differentiate them from explicit inputs and outputs. Graphs comparing **b**, the cumulative energy demand, **c** global warming potential, and **d** cumulative water use of the different graphene synthesis methods.

Our prospective cradle-to-gate LCA considered the impacts resulting from the mining or preparation of raw materials (cradle) and all synthetic processes that would occur at a factory (gate) for production of 1 kg of graphene powder as the final material from three synthetic pathways: (1) FJH from plastic waste feedstocks as described here, (2) ultrasonication solution phase exfoliation of graphite^60^, and (3) chemical exfoliation of graphite to form graphene oxide using modified Hummer’s process followed by chemical reduction using hydrazine^61^. A cradle-to-gate LCA does not consider the use of materials nor their disposal (grave). Process flow diagrams for each pathway are shown in Fig. 5a. A cut-off approach was employed in the case of the plastic waste feedstock used in the FJH process whereby the burdens associated with the virgin polymer production are attributable to the prior product. Thus, the plastic contained in the shredder residual is treated as a waste product with no associated burdens. Due to environmental regulations and general waste management strategies in developed countries as detailed in the introductory literature review, the ELV-WP already is depolluted, dismantled, and shredded as part of metal recovery prior to generally being landfilled. The ELV-WP is therefore an incidental output of the metal recovery process. Using the cut-off approach, the emissions, energy use, and water use of the depollution, dismantling, and shredding steps are affiliated with prior vehicle disposal and are not considered in the present LCA or LCI.

We have assumed that the use and end-of-life phases for the graphene post-synthesis will be identical regardless of the production pathway, thus we constructed the study as a process centered cradle-to-gate LCA. However, this simplifying assumption might need to be refined in future research. As discussed previously, the turbostratic layering of the produced ELV-WP-FG provides for both easier exfoliation and a much higher factor of dispersibility in solution phase by sonication than existing commercial graphene. These characteristics will lead to less energy being used in dispersion and lower loadings of graphene to be used in composite applications.

### LCA results

The results of the prospective LCA suggest that FJH results in substantial reductions in energy, greenhouse gas emissions, and water use relative to both physical and chemical exfoliation (Fig. 5b–d). Compared to an ultrasonication synthetic method, FJH was shown to reduce CED by 88%, reduce GWP by 85%, and decrease CWU by 93%. Similar improvements were observed when comparing FJH to chemical exfoliation techniques: FJH afforded an 80% reduction in CED, decreased GWP by 80%, and reduced CWU by 97%. These large improvements in sustainability are obtained in both raw material input requirements and process considerations. Interestingly, for the chemical exfoliation process, the largest contributing material input to CED (39%), GWP (42%), and CWU (36%) was the H_3_PO_4_. Clearly, aqueous media required for reaction and rinsing steps also contributed 40% to CWU for the chemical exfoliation synthetic route. The process energy inputs during the chemical exfoliation were found to be minor for each category as heating temperatures and durations are low. Although treatment of process outputs were not considered in this prospective LCA, it should be noted that H_2_SO_4_ containing KMnO_4_ is extremely hazardous and a difficult waste streams to manage^61^.

For the sonication method of exfoliation, the material contributing the most to the CED (40%), GWP (26%), and CWU (54%) is the ethanol solvent used, despite the assumption that 97% is recovered and reused. Rinsing solvents are again larger contributors to the CED, GWP, and CWU than the graphite precursor. The specifics of sonication-based exfoliation methods are much less agreed upon than chemical exfoliation and reduction^60,61^. In sonication-based exfoliation methods, it is clear from our LCA that solvent choice, surfactant choice, sonication duration, solvent recovery, and assumed dispersion concentration can each impact the CED, GWP, and CWU by large amounts. For the FJH synthetic route, over 96% of impacts across all three categories are associated with the electricity used during flashing. This demonstrates that through simple use of renewable energy sources, the GWP and CWU might be further decreased.

Results of our analysis for chemical and ultrasonication pathways is comparable to previous LCA findings (Supplementary Table 2), and direct comparisons to other graphene LCAs is given in the Supplemental Information under the heading of “Supplemental Discussion of the LCA CED Values”. The lack of standardization across the graphene industry indicates that this LCA is subject to refinement in subsequent studies. Potential improvements in the FJH process including yield improvements might further reduce CED, GWP, and CWU. Volatile gaseous effluents resulting from the LC-FJH process might provide valuable co-products and further contribute to the environmental proposition of the FJH upcycling of polymers into FG. We acknowledge that exfoliation-based strategies also have improvement potential. The need to account for disposal methods to manage byproducts from all synthetic processes will likely increase burdens by widely varying amounts. Further detailed environmental analysis of graphene synthesis will serve to drive improvements and advancement.

### Continuous upcycling of graphene enhanced polyurethane foams

Beyond lowering energy, climate, and water burdens, an additional sustainability goal for product systems is preserving material utility. Establishing circular material flows in recycling processes is an important sustainability strategy for industrial products. The key characteristic in closed loop recycling processes is the maintenance of original materials properties, which allows for recycled material to displace virgin material^59^. Upcycling is the process of converting a material into a new resource of higher quality, value and increased functionality. Both approaches avoid material downcycling, which can narrow the potential uses for recycled materials to less valuable applications and thus continue the demand for virgin materials for higher value applications.

**Fig. 6:**
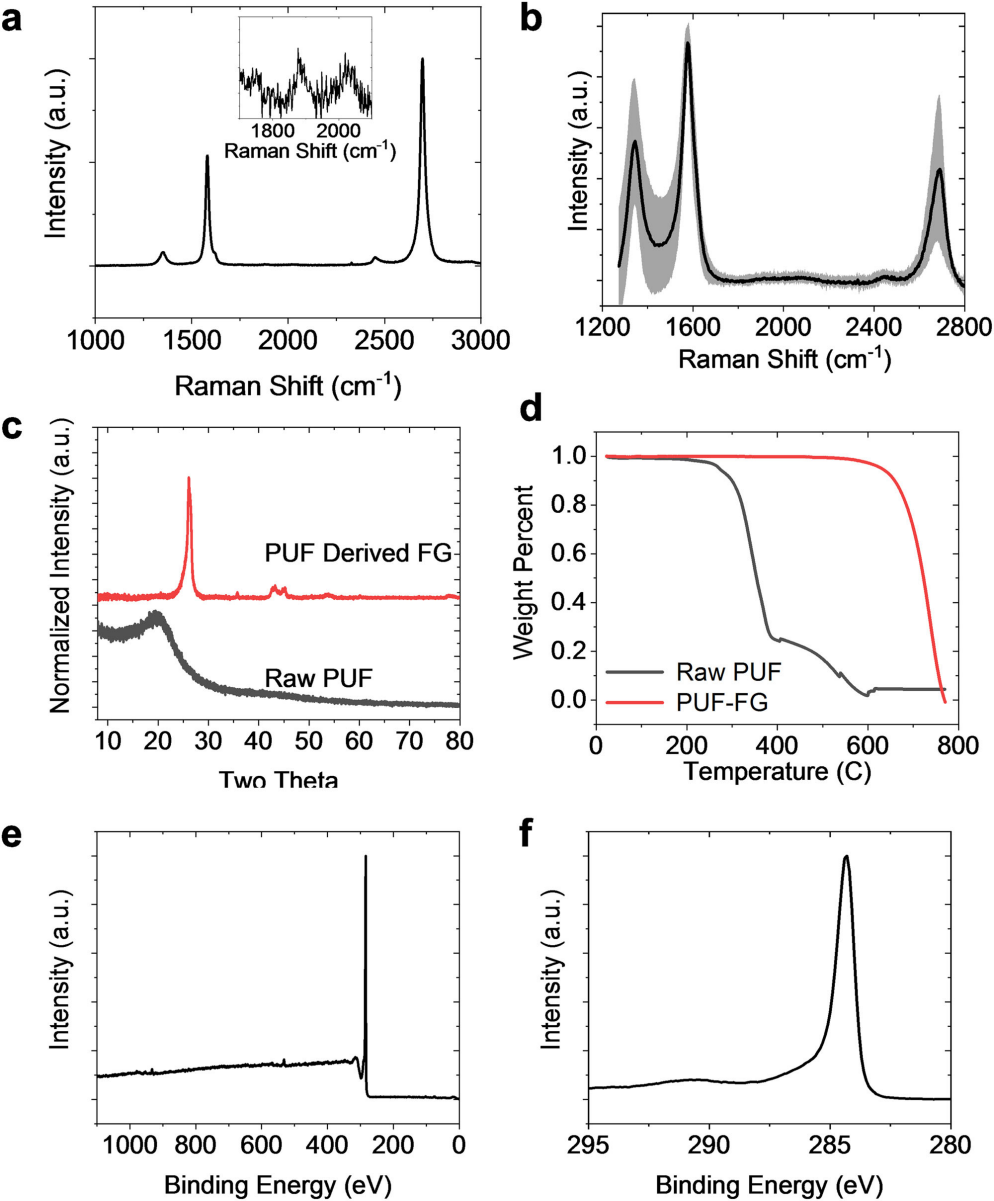
Characterization of continuously recycled ELV-WP-FG-PUF composites showing FG can be synthesized from FG-containing composite materials. Raman characterization showing (**a**) an exemplary spectrum with expanded inset showing turbostratic indicators and (**b**) average spectra of 100 spots with the shaded region representing the standard deviation. **c** Powder XRD, **d** TGA under air flow with a purge rate of 80 ml min^−1^, and **e**, **f** XPS survey spectrum and C1s high-resolution spectrum of the FG derived from the PUF composites.

The FJH process has already been shown to upcycle a waste material into a higher-value resource. To demonstrate that the FJH process of ELV-WP can be repeatedly used to continuously recycle the same waste stream effectively, the ELV-WP-FG-PUF composites were again converted to graphene using FJH. Recycling of polymer composites containing nanomaterials remains a challenge for traditional recycling technologies, giving enhanced value to these findings. The FG was characterized (Fig. 6) and shows high quality bulk FG can again be produced from the ELV-WP-FG-PUF composite. No obvious difference in quality of the FG samples is observed between the ELV-WP starting material or ELV-WP-FG-PUF starting material. Raman spectra, both single spots as well as the average of 100 spots, demonstrate the good quality of the FG. Powder XRD shows conversion of the largely amorphous polymer precursor, while TGA demonstrates a high thermal stability and purity of FG. XPS survey scans show a carbon content of 95.6%, with 2.6% oxygen content and 1.8% oxygen content, with a lack of any other major contamination. High-resolution scans of the C1s peak disclose the binding to be solely sp^3^/sp^2^ bonding. High-resolution scans of the N1s region show pyridinic and pyrrolic binding of the N present, suggesting that the FG produced from the PUF (containing 6% N) is N-doped high quality graphene (Supplementary Fig. 14). Therefore, re-flashing of the FG-composites afford new FG of the same quality for further incorporation into new composites.

## Conclusion

In conclusion, high quality turbostratic FG is prepared from ELV-WP using FJH, a simple and rapid solvent-free synthetic method. This was accomplished through design and assembly of an LC/HC FJH reactor with dual LC and HC capabilities. The graphene was then incorporated into PUF composites affording improvements in physical and mechanical properties. The interface between ELV-WP-FG and PUF was studied to better understand the composites using DSC and cross-sectional SEM imaging. Continuous upcycling was further demonstrated through conversion of the ELV-WP-FG-PUF composites back into FG using the FJH process. Finally, a prospective LCA was conducted to allow for approximate comparison between the FJH process and traditional graphene synthetic methods demonstrating that large improvements can be made in CED, GWP, and CWU.

## Methods

### Chemicals and materials

Authentic Ford F-150 ELV-WP, which is a broadly mixed source of plastic types, was kindly provided by Ferrous Processing and Trading (Detroit, Michigan, United States) and was ground to a fine powder via an electric hammer mill (CGoldenWall, Model DF-15) before use. This ELV-WP derived from Ford F-150 pick-up trucks was used without any further processing such as sorting or rinsing. Metallurgical coke (metcoke) was provided by SunCoke Energy (Granite City, Illinois, United States) and used without further purification. A typical cost for metcoke is ~$400 ton^−1^.

### Synthesis of ELV-WP-FG

A custom-made LC/HC FJH reactor was built for the conversion of ELV-WP into graphene. A circuit schematic and further details are shown in Supplementary Fig. 1. A LC rectified AC, longer duration heating (LC-FJH) is used to carbonize the ELV-WP (1–25 A, over 15–20 s, yielding 30% mass recovery). Then, shortly after, in the same reactor, a DC HC, short duration heating (HC-FJH) is used to convert the carbonized ELV-WP to graphene (200 A from 104 mF capacitors charged to 150 V, discharged in 500 ms). ELV-WP (2.6 g) was mixed with 5 wt% metcoke (0.13 g) during the hammer milling process to increase the conductivity of the sample. The ground material was compressed in a quartz tube and sandwiched between copper wool and graphite electrodes (graphite in contact with the sample) to conduct current through the sample that had an initial resistance of ~500 Ω. The loaded quartz tube was enclosed in a plastic vacuum desiccator (50 Torr) to trap and remove sublimated impurities and outgases. A final resistance of 1 Ω resulted after the sequential LC-FJH and HC-FJH processes, recovering 19–24% of the original plastic weight as FG powder. After the FJH, the newly formed graphene was removed from the quartz tube and used without further purification or treatment.

### Fabrication of FG-polyurethane foam (ELV-WP-FG-PUF)

PUF was made from a reaction of petroleum polyol and diisocyanate using FG powder as a filler. The formulation begins with polyol combined with a cell opener, surfactant, crosslinker, catalysts, and a blowing agent. The chemicals used are outlined in Supplementary Table 1. Graphene powder was added to the mixture and mixed for 3 min at 1500 RPM to generate a homogenous-looking mixture of the filler. After mixing, the diisocyanate was added to the mixture and mixed for 12 s. The mixture was moved to a 30.5 × 30.5 × 5.1 cm^3^ mold and heated to 65 °C for 7 min. Chem-Rend PU-11331 was used as a mold-release. The foam sample was then post-cured in an oven at 65 °C for 30 min and then rested at room temperature for a minimum of 24 h. PUF samples were prepared and tested with a four different graphene loading levels, 0.01, 0.025, 0.05, and 0.1%, to determine the effect of the graphene on the mechanical, thermal and other physical properties.

### Life cycle assessment

A prospective LCA was conducted for FG produced from FJH and compared with graphene produced from graphite exfoliated by sonication or by oxidation to graphene oxide followed by chemical reduction. The study goal was to compare the cradle-to-gate impacts among these three alternative graphene synthesis pathways and thus graphene use and disposal was excluded. A functional unit of 1 kg of graphene powder was considered. Powdered graphene rather than solution phase dispersed graphene was modeled since it is of wider utility industrially and lighter to ship. The CED, GWP over a 100-year timescale, and CWU were evaluated. Process input and output data for sonication and chemical pathways were based on literature^60,61^. Material transportation or waste stream disposal/remediation were outside the scope of this limited study. Background data was principally sourced from Argonne National Laboratory’s GREET model including both GREET.Net software and spreadsheet models^56,57,62^. A detailed spreadsheet of process inputs and outputs as well as inventory and impact calculations can be found in the attachment Supplementary Data 1.

### Characterization

All Raman spectra of FG were collected from samples, ground by mortar and pestle and not exposed to solvent. A Renishaw inVia Raman microscope outfitted with a 5 mW 532 nm laser was used, with ×50 optical objective lenses to collect high magnification spectra. All XRD spectra were collected of samples ground by mortar and pestle and not exposed to solvent. A Rigaku D/Max Ultima II Powder XRD 6 s were used to collect XRD patterns. A scan width of 0.05° per step and scan rate of 0.5° min^−1^ was used from 3° to 90°. Zero background sample holders were used. TGA thermograms were collected of samples ground by mortar and pestle and not exposed to solvent. A Q-600 Simultaneous TGA/DSC from TA Instruments was used. Alumina pans were used at a heating rate of 10 °C min^−1^ up to 780 °C. Atmospheric air at a flow rate of 80 ml min^−1^ was used to continuously purge the sample chamber. To determine the concentration of FG present in dispersed aqueous solutions, varying amounts of finely ground FG were added to a 1 wt% Pluronic-F127 aqueous solution. The solutions were cup horn ultrasonicated for 10 min at 25 °C, then centrifuged at 1000 RCF for 20 min. The supernatant was then diluted 200x and the absorbance measured at 660 nm. An extinction coefficient of 6600 l g^−1^ m^−1^ was used. An identical procedure was used to make commercial graphene dispersions. SEM images were taken with a FEI Helios Nanolab 660 Dual Beam SEM System. Low-voltage (1 keV) scans were taken of the PUF composites to minimize charging. XPS data were collected with a PHI Quantera SXM Scanning X-ray Microprobe with a base pressure of 5 × 10^–9^ Torr. Survey spectra were recorded using 0.5 eV step sizes with a pass energy of 140 eV. Elemental spectra were recorded using 0.1 eV step sizes with a pass energy of 26 eV. All of the XPS spectra were corrected using the C 1 s peaks (284.8 eV) as reference. TEM images were collected using a JEOL 2100F TEM system using samples of ELV-WP-FG briefly sonicated in ethanol, then drop cast on lacy carbon grids and allowed to air dry. An accelerating voltage of 200 kV was used. ASTM 3574-08 (Test A), ASTM 3574-08 (Test L), ASTM 3574-08 (Test C), ASTM 3574-08 (Test E) and ASTM D 624 (Die C) were used for apparent density, wet compression set, compression force deflection, tensile testing, and tear strength, respectively. At least six replicates were used for each measurement. The Tg was measured on a TA DSC 2500 DSC, using a sealed Tzero aluminum pan and lid. The foam samples of ~5 mg were investigated in nitrogen atmosphere from −90 to 100 °C, at a heating rate of 10 °C min^−1^ and a nitrogen flow rate of 50 ml min^−1^. The acoustic properties were found using a B&K Type 4206 two-microphone impedance tube (100 mm samples from 50–1600 Hz and 29 mm samples from 500–5000 Hz) to determine the plane wave absorption. The Transfer Function Method, ISO 10534-2 is used to calculate the Reflection factor and from that the Absorption and the Normal Specific Acoustic Impedance are calculated.

### Supplementary information

Supplementary Information

Supplementary Data 1

Supplementary Data 2

Description of Additional Supplementary Files

## Data Availability

The data used during this study are available in the manuscript and Supplementary Information. We have uploaded the source data to the Zenodo database, accessible at: 10.5281/zenodo.6335713. The source data in excel format is also uploaded as Supplementary Data 2.
