# Supplementary material for: Upcycling end-of-life vehicle waste plastic into flash graphene
Source: Commun Eng. 2022 May 26;1:3. doi: 10.1038/s44172-022-00006-7 (PMC10955767; doi:10.1038/s44172-022-00006-7)
Supplement: Supplementary file 1 — Supplementary Information [file 44172_2022_6_MOESM1_ESM.pdf]

Supplementary Material

Upcycling End-of-life Vehicle Waste Plastic into Flash Graphene

Kevin M. Wyss,[1] Robert D. De Kleine,[2] Rachel L. Couvreur,[2] Alper Kiziltas,[2] Deborah F. Mielewski,[2] James M. Tour[1,3,*]

[1]Department of Chemistry, Rice University, 6100 Main Street MS 222, Houston, TX 77005 USA.

[2]Research and Innovation Center, Ford Motor Company, 2101 Village Rd., Dearborn, Michigan, 48124, USA.

[3]Smalley-Curl Institute, NanoCarbon Center, Welch Institute for Advanced Materials, Department of Materials Science and Nanoengineering, Department of Computer Science, Rice University, 6100 Main Street MS 222, Houston, TX 77005 USA.

*Email: tour@rice.edu

Supplemental Figures:

a)

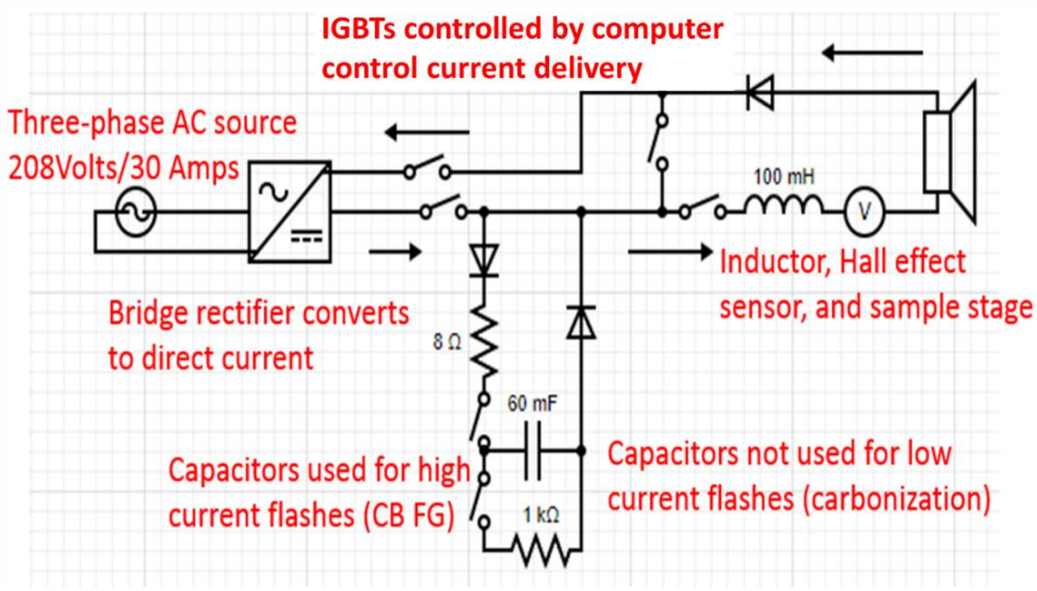

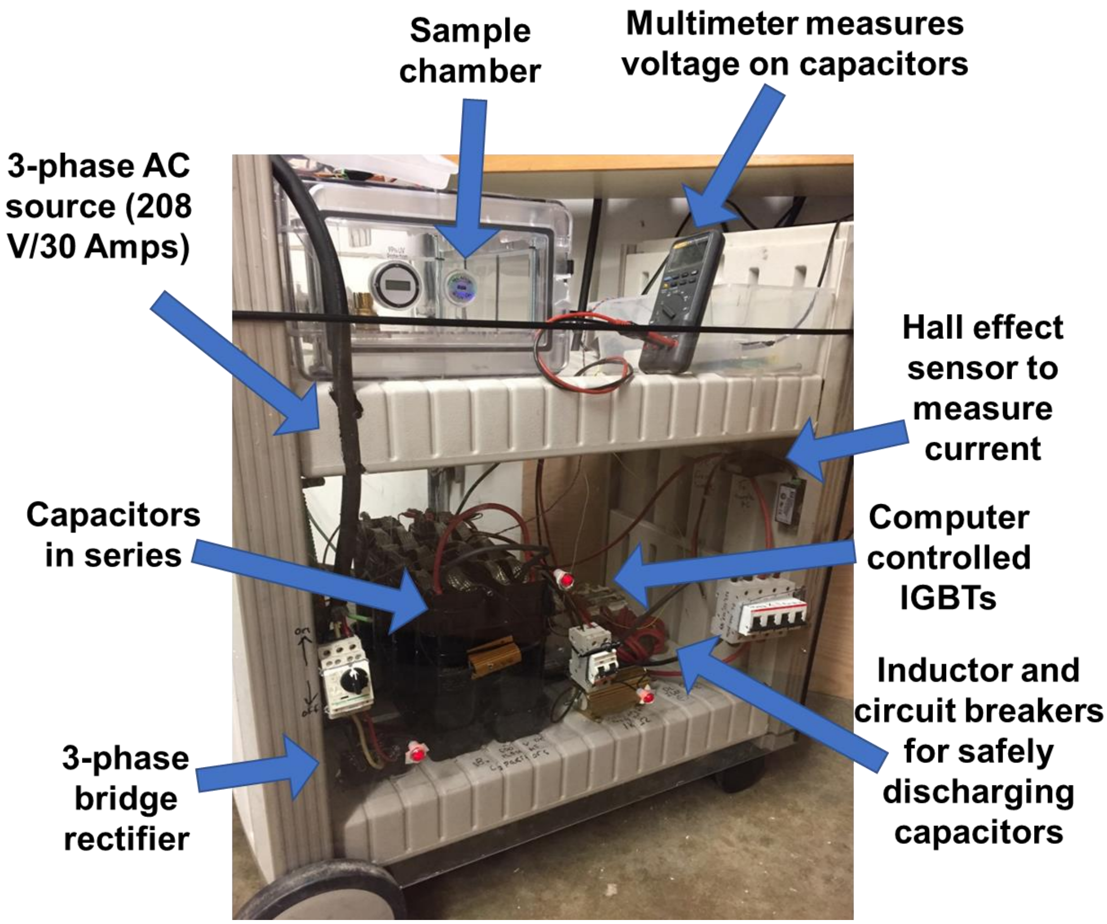

Supplementary Figure 1: a Circuit schematic for the LC/HC FJH system with components labelled. **b** Photograph of LC/HC FJH station built on a plastic cart.

Supplemental Discussion: Flash Joule heating safety considerations and design

CAUTION: There is a risk of electrical shock or even electrocution so the safety features previously discussed (Nature, 577, 647-651, 2020) should be implemented. The following list (from Nature, 577, 647-651, 2020) is not intended to be comprehensive but demonstrative of the protocols needed to minimize risk.

1. Enclose or carefully insulate all wire connections.

2. All connections, wires and components must be suitable for the high voltages and currents.

3. Be aware that component failure could cause high voltage to appear in unexpected places, such as heat sinks on the switching transistors.

4. Control wires should have opto-isolators rated for high voltage.

5. Provide a visible charge indicator. A 230 V clear glass incandescent light bulb is a good choice as the glow on the filament also provides an approximate indicator of the amount of charge on the capacitor bank. Bright light = danger!

6. Do not use toggle switches with metal toggles. If an arc develops, the metal toggle could become charged. Use only properly rated circuit breakers.

7. One hand rule. Use only one hand when working on the system, with the other hand not touching any grounded surface.

8. Install bleed resistors in the range of 100,000 ohms on each capacitor so that charge will always bleed off in ~1 h.

9. Provide a mechanical discharge circuit breaker switch connected to a power resistor of a few hundred ohms to rapidly bleed off the capacitor charge.

10. Provide a "kill" circuit breaker switch to disconnect the sample holder from the capacitor bank.

11. Provide an AC disconnect circuit breaker switch.

12. Post high voltage warning signs on the apparatus.

13. Use of circuit breakers as switches. Circuit breakers have built-in arc suppression that can interrupt 1000 amps or more. Conventional switches do not have such a high level of arc suppression and can burn out or weld closed due to the high current pulses.

14. Use circuit breakers rated for DC voltage. Most AC circuit breakers have a DC rating ½ the voltage or less, since DC arcs are much more difficult to suppress. Circuit breakers designed for DC solar power systems are a good choice.

15. When choosing circuit breakers, choose by the time curves typical for 0.1 s, rather than the steady state current rating. K-type DC circuit breakers will have ~10x higher trip current at 0.1 s compared to their rated current, and Z-type breakers will have ~4x higher trip current at 0.1 s. This "delayed trip" designed into most circuit breakers will allow much higher pulse currents than the steady state rating of the breaker.

16. Include a small amount of inductance in the discharge circuit to limit the rise time to a millisecond or more. Extremely fast discharges can damage components and cause RF interference with other lab apparatii.

17. Keep in mind that the system can discharge many thousands of Joules in milliseconds, which can cause components such as relays or even capacitors to explode. These components should be enclosed to protect against both high voltage and possible flying debris.

18. Keep a voltmeter with high voltage test leads handy at all times. When working on the capacitor bank, always check the voltage on each. A broken wire or loose connection could leave the capacitor in a charged state.

19. Wear thick rubber gloves when using the apparatus to protect from electrocution.

20. All users should be properly trained by an experienced electrical technician.

21. Welder’s glasses should be worn to minimize eye damage risk by the bright emitted light resulting from the flash (IR and visible photons can cause eye damage).

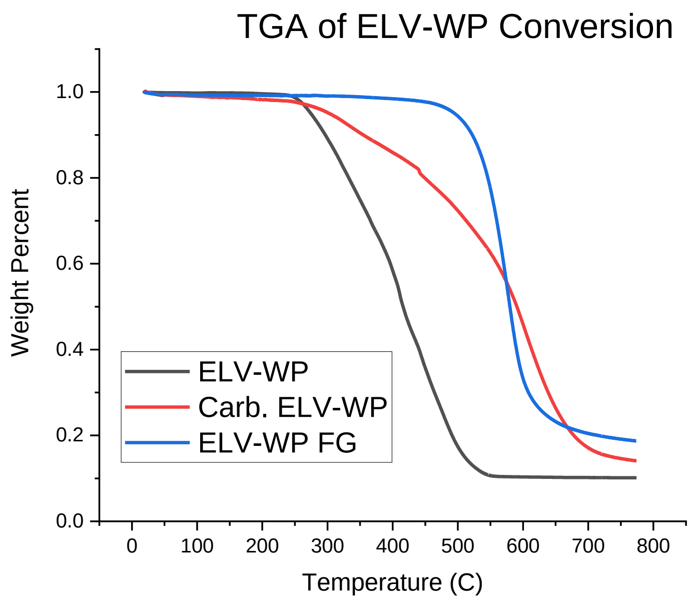

Supplementary Figure 2: TGA of ELV-WP, carbonized ELV-WP (after LC step), and ELV-WP-FG.

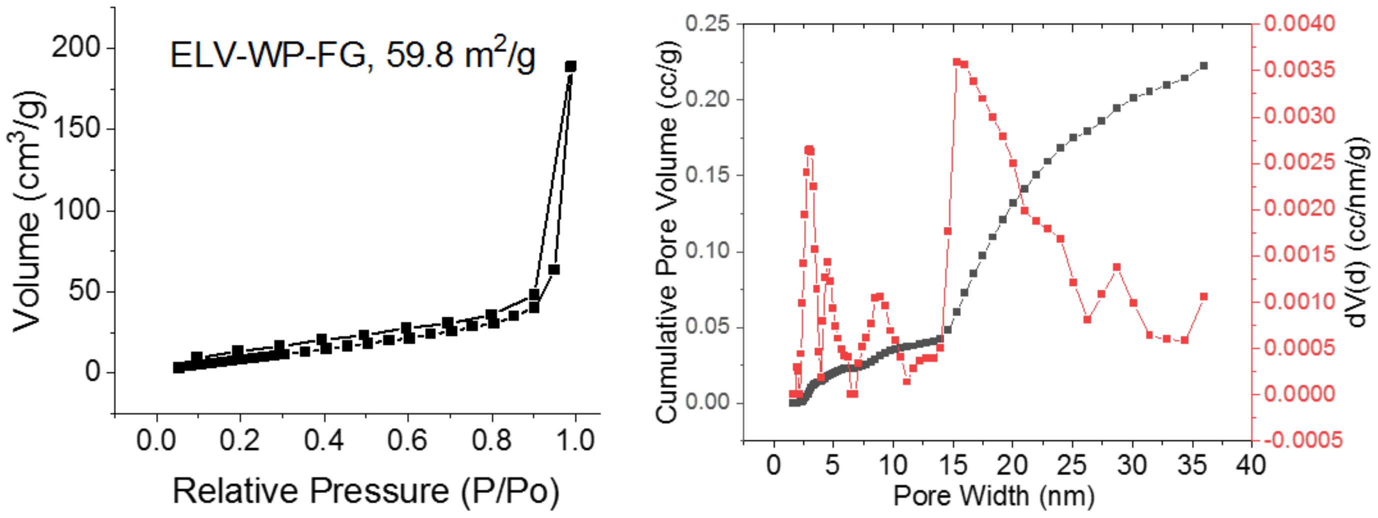

Supplementary Figure 3: a, BET surface area analysis gas adsorption isotherm and **b,** DFT calculated pore size.

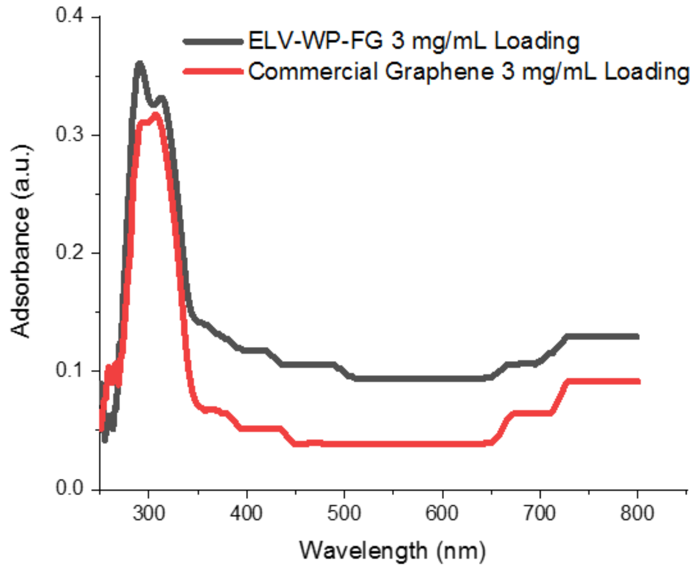

Supplementary Figure 4: UV-Vis spectra showing the highest concentrated dispersion of commercial graphene and ELV-WP-FG.

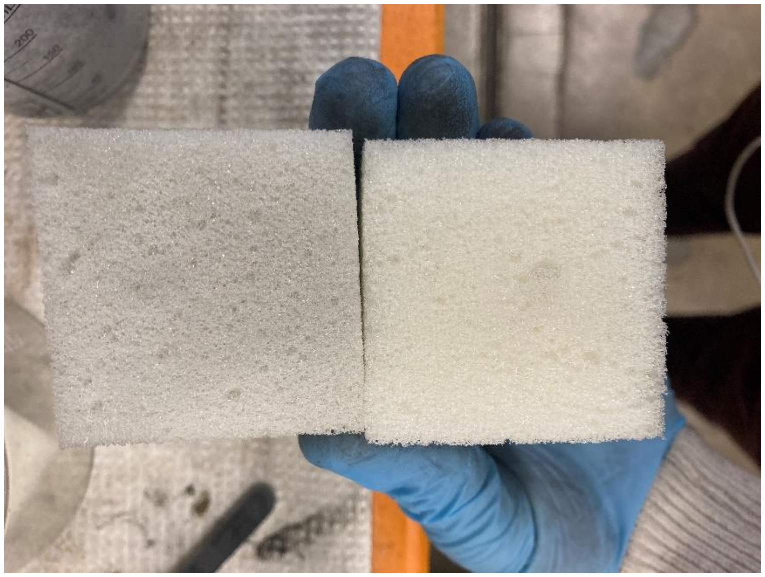

Supplementary Figure 5: Optical images of the ELV-WP-FG-PUF composites. **a**, A sample with 0.1% ELVWP-FG added; **b**, a control sample with no ELV-WP-FG added.

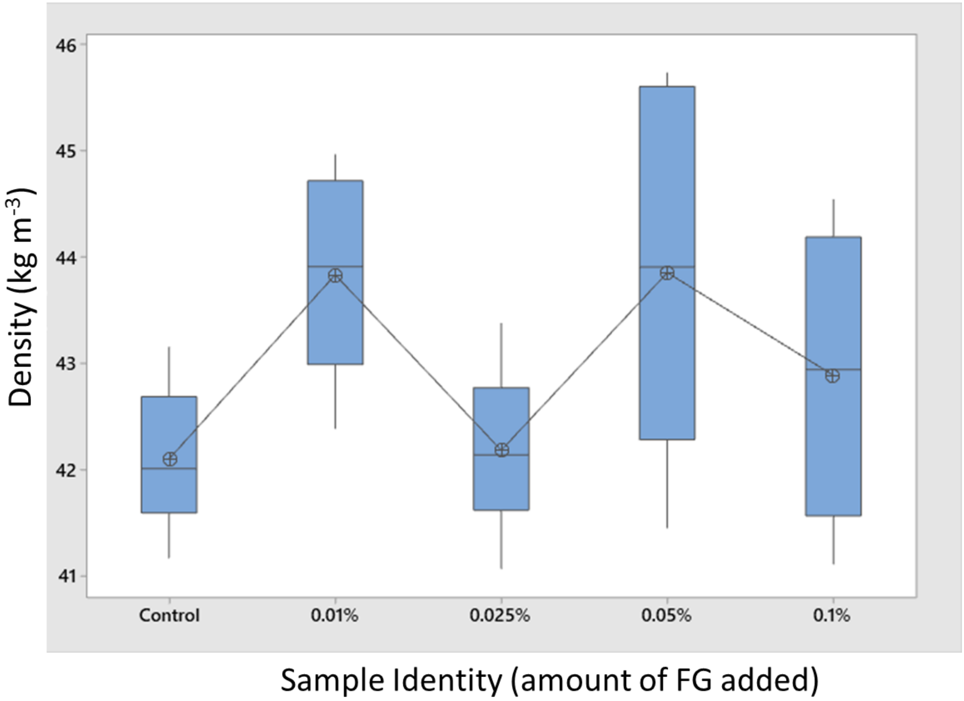

Supplementary Figure 6: Box and whisker plots showing the density of ELV-WP-FG-PUF composites as ELV-WP-FG content is varied, N = 6. The interquartile range is shown by shaded 'box', while the maximum and minimum are shown by the 'whiskers'.

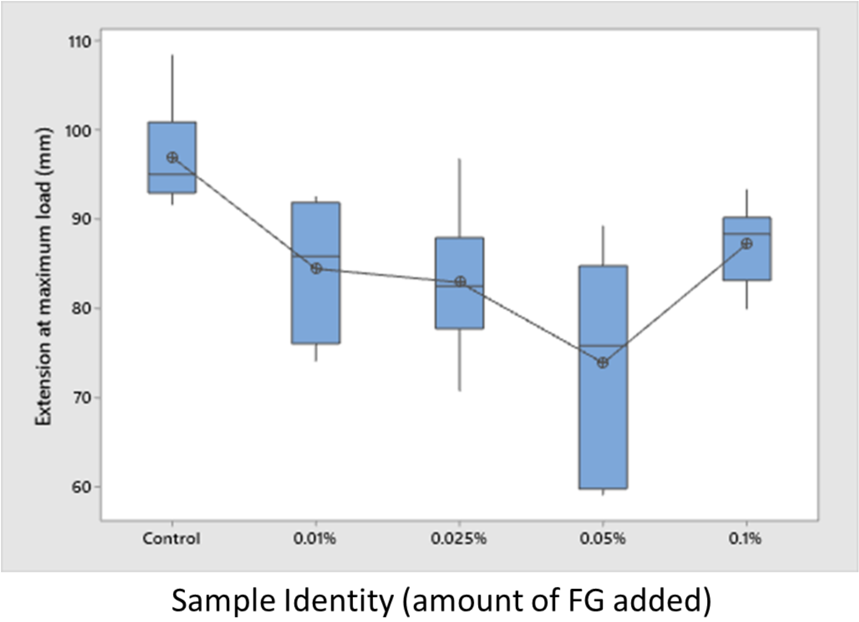

Supplementary Figure 7: Box and whisker plots showing the ELV-WP-FG-PUF composites extension at max load as ELV-WP-FG content is varied, N = 6. The interquartile range is shown by shaded 'box', while the maximum and minimum are shown by the 'whiskers'.

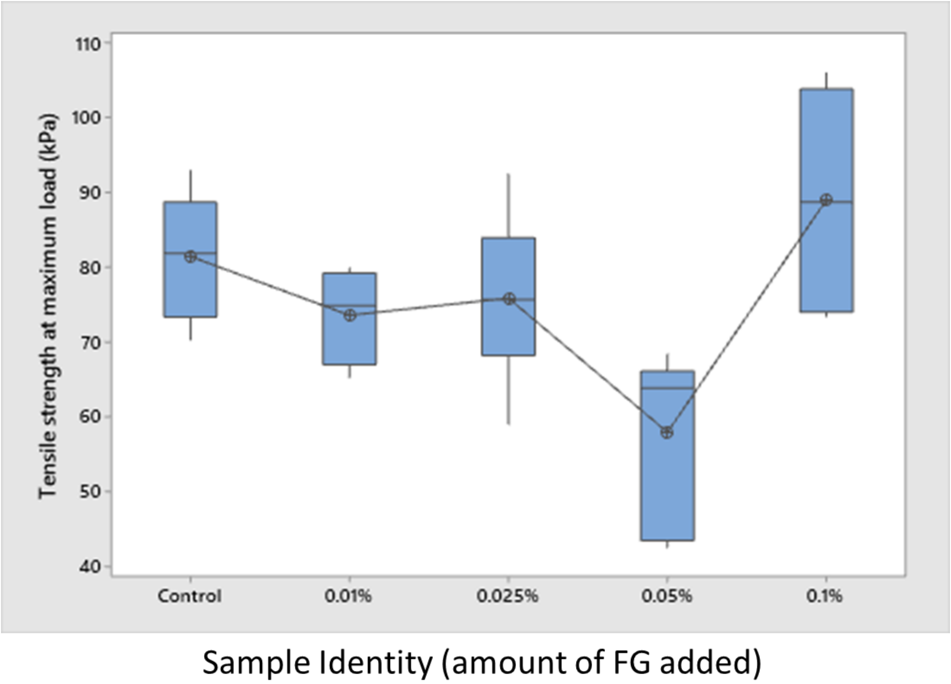

Supplementary Figure 8: Box and whisker plots showing the ELV-WP-FG-PUF composites tensile strength at max load as ELV-WP-FG content is varied, N = 6. The interquartile range is shown by shaded 'box', while the maximum and minimum are shown by the 'whiskers'.

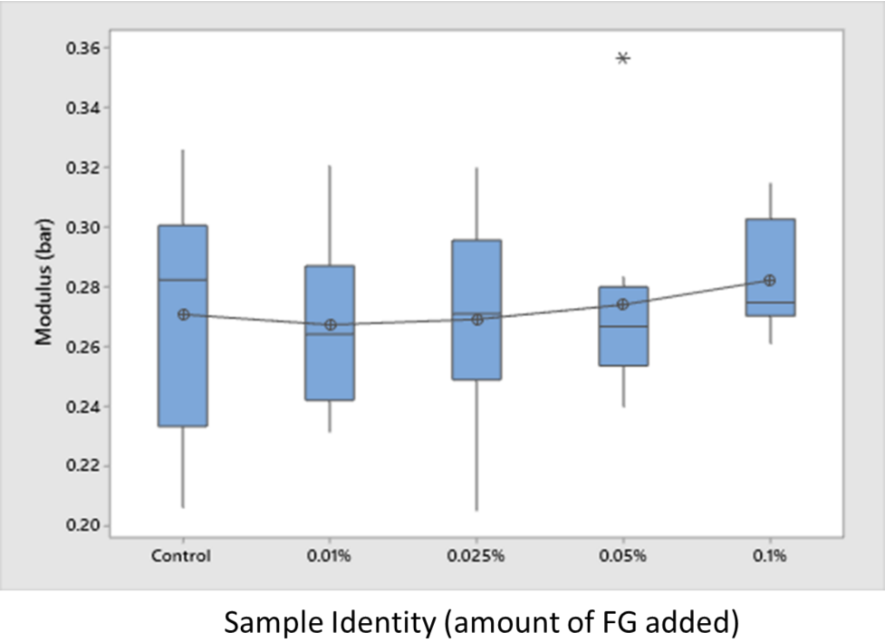

Supplementary Figure 9: Box and whisker plots showing the ELV-WP-FG-PUF composites compressive modulus, as ELV-WP-FG content is varied, N = 6. The interquartile range is shown by shaded 'box', while the maximum and minimum are shown by the 'whiskers'.

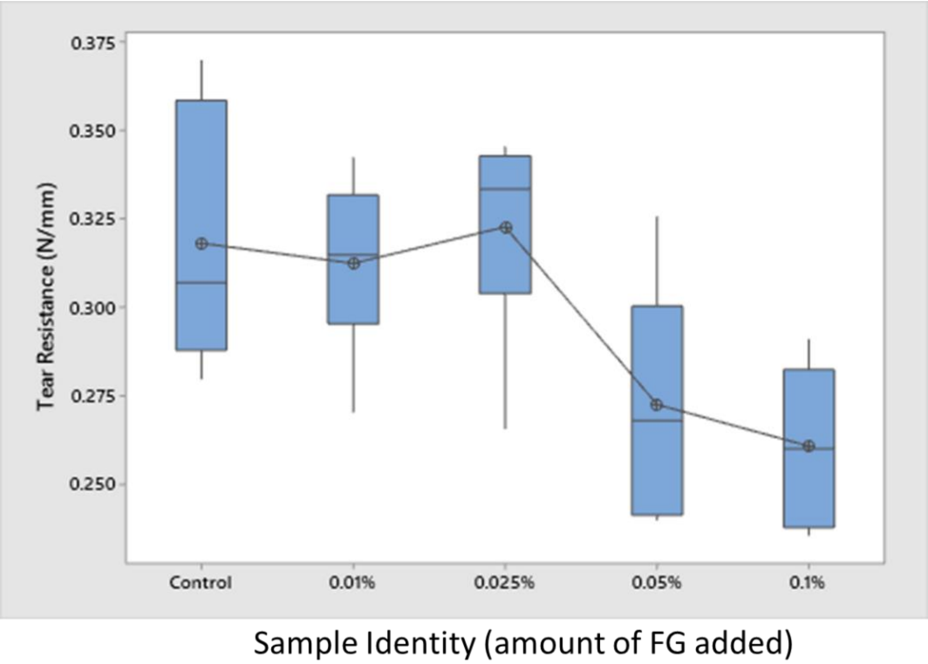

Supplementary Figure 10: Box and whisker plots showing the ELV-WP-FG-PUF composites tear resistance as ELV-WP-FG content is varied, N = 6. The interquartile range is shown by shaded 'box', while the maximum and minimum are shown by the 'whiskers'.

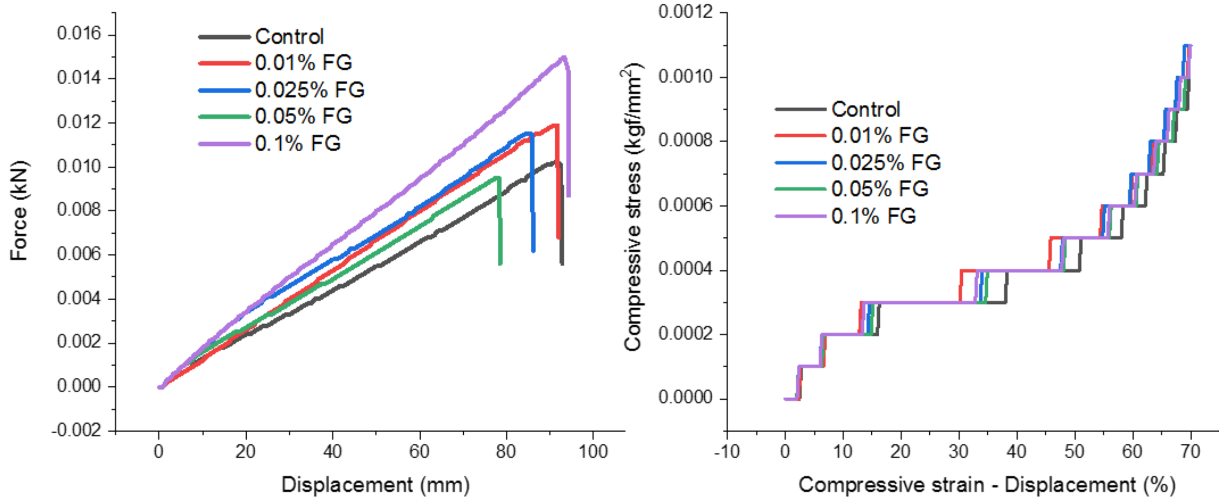

Supplementary Figure 11: Representative compression and tension curves for each sample from the Instron testing.

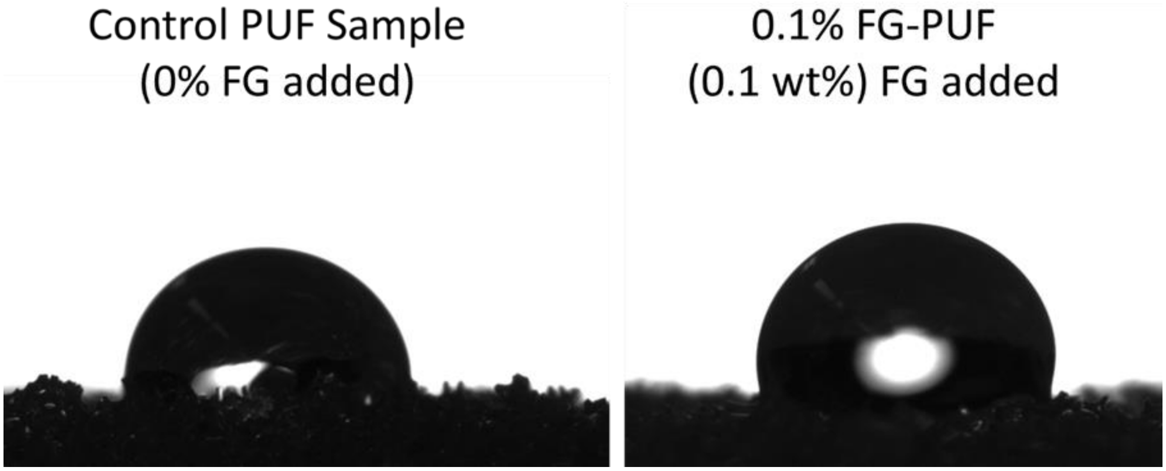

Supplementary Figure 12: Water contact angle goniometry comparing the contact angle of (left) raw PUF with (right) 0.1% added FG-PUF.

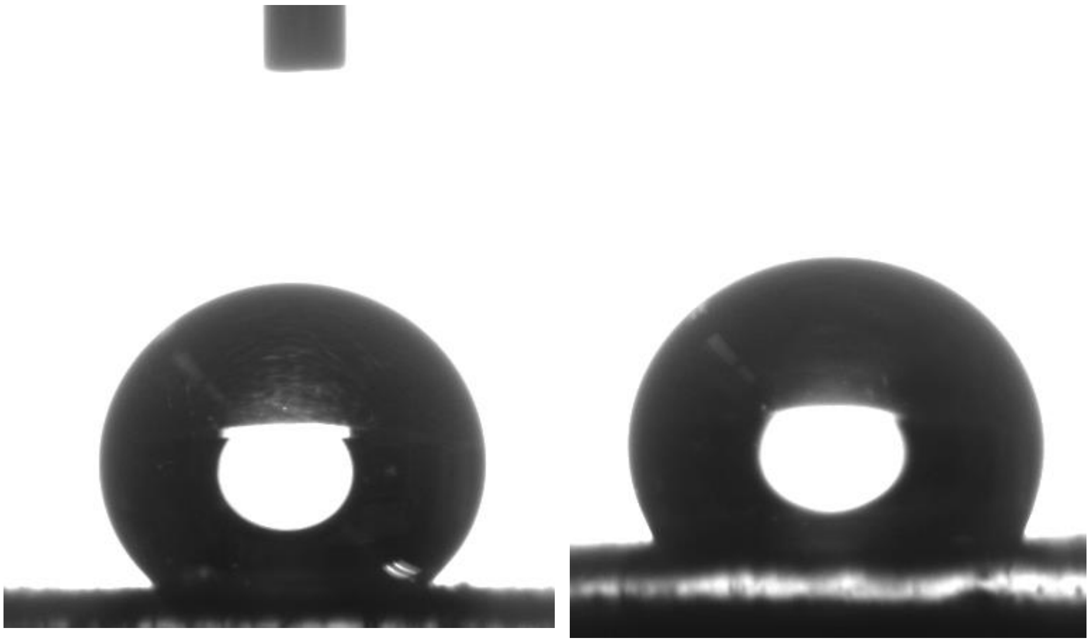

Supplementary Figure 13: Water contact angle goniometry comparing the contact angle of ELV-WP-FG (left) and PUF derived FG (right) films formed by vacuum filtration.

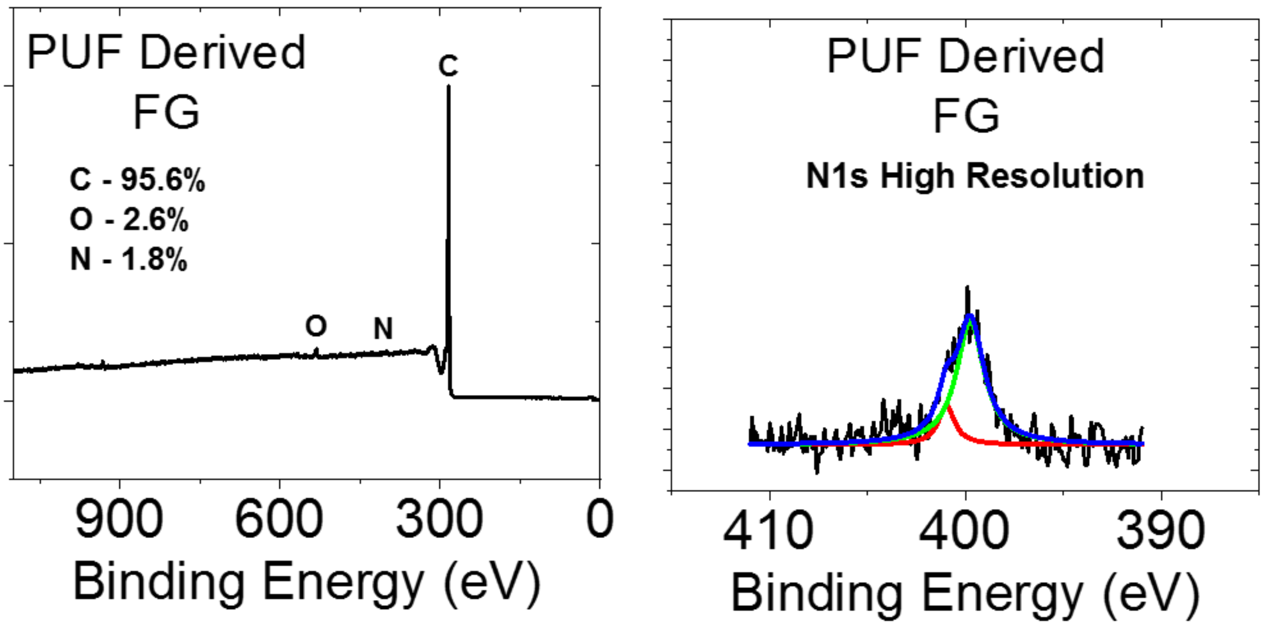

Supplementary Figure 14: XPS analysis of the PUF derived FG (let), with high resolution scans of the N1s region also shown on the right.

Manufacturer	Chemical	Component Type
Dow Chemical Co. (Midland, MI)	Voranol 4701	Petroleum Polyol
Lambent Corporation (Gurne, IL)	Lumulse POE (26) GLYC	Cell Opener
Lambent Corporation (Gurne, IL)	Tegostab B4690	Surfactant
Sigma Aldrich (St. Louis, MO).	Diethanolamine (DEA)	Cross Linker
Momentive Inc. (Waterford, KY)	Niax A300	Catalyst
Momentive Inc. (Waterford, KY)	Niax A1	Catalyst
N/A	Deionized Water	Blowing Agent
Huntsman International LLC (Auburn Hills, MI)	Rubinate 7304	Diisocyanate

Supplementary Table 1: The identities, manufacturers, and types of chemicals used in the synthesis of PUF matrices.

	Cumulative Energy Demand (CED) (MJ kg^{-1} graphene)	Graphene Production Method	Notes
This Work:	262	FJH	Assumes virgin plastic feedstock, not upcycling of existing waste plastic
Studied in this work:	2351	Sonication	Ethanol with surfactant, 1 mg/mL graphene concentration, 97.5% solvent recovery, rinse removal of surfactant
Studied in this work:	1334	Chemical	Modified Hummer's method (Marcano et al) followed by hydrazine reduction
Serrano-Lujan et al. 2019[3]	20,070	Chemical	Includes waste stream management
Arvidsson et al. 2014[4]	1,100	Chemical	Solution phase graphene product
Pizza et al. 2014[5]	1,879	Microwave	Powder graphene nanoplatelet product
Cossutta et al. 2017[6]	3,140	Electrochemical	Electrolysis of water with graphite electrode and KOH, filter, sonicate
Cossutta et al. 2017[6]	1640-3290	Chemical	Hummer's method and variations, with both chemical and thermal reduction methods considered
Arvidsson et al. 2014[4]	470	Sonication	Solution phase graphene product, no surfactant used, assumes solvent recovery but does not factor into CED, no process graphene concentration or solvent volume specified

Supplementary Table 2: A comparison of CED data from recent LCAs of graphene synthetic methods.

Supplemental Discussion of the LCA CED Values. As shown in **Supplementary Table 2**, the CED values from this study generally agree well with LCAs that have previously been done. The clear outliers are the FJH method (discussed in detail in the manuscript), the study by Serrano-Lujan *et al*. in 2019, and the study on sonication by Arvidsson *et al*. in 2014. The Serrano-Lujan study includes disposal and waste stream management as well as some production stream

infrastructure/hardware. Due to the volume and identity of chemical waste streams produced, from the strong oxidizing agents and reducing agents, it is not surprising that consideration of waste streams greatly increased their reported CED. Concerning the results disclosed in the 2014 Arvidsson publication, they determine that sonication uses 470 MJ kg^{-1} of graphene solution synthesized. However, their LCA has some key differences and is therefore difficult to directly compare to our findings. First, their functional unit for the study is 1 kg of graphene in solution, whereas our functional unit is 1 kg of graphene powder. Most noticeably, at no point in the manuscript is the assumed amount of solvent needed or assumed graphene dispersion concentration disclosed, and no surfactant is used. It is well-documented that pristine graphene concentrations in solvent systems with no surfactant are extremely low (see: Y Hernandez *et al.*, where they reported concentrations of 0.01 mg mL^{-1}).[7] Despite this, Arvidsson *et al.* use diethyl ether with no surfactant as the sonication media in their LCA, citing a patent by Zhamu *et al*.[8] Zhamu *et al.* assert that graphene concentrations between 1 mg mL^{-1} and 10 mg mL^{-1} can be reached in these surfactant-free polar solvent systems, yet no data supporting this claim (such as UV-Vis absorption or solution phase Raman spectroscopy) is given in the patent, and to our knowledge no literature exists supporting these claims. Further, assuming that such high concentrations are indeed able to be achieved during sonication, it is unclear how the graphene will not re-aggregate to graphite in the solvent system following sonication. Further, a beginning grain size of 20 μm for the graphite precursor is required by Zhamu *et al.*, but no graphite milling step is discussed by Arvidsson *et al*. Further, in their sensitivity analysis, they demonstrate that assumption of solvent recovery can greatly improve the calculated CED and environmental impacts. However, at no point does Arvidsson *et al.* discuss how they would recover the solvent, or how this recovery process would impact the CED. Lastly, no Raman spectroscopy is given to

characterize the quality of, or even confirm the presence of, graphene produced through this surfactant-free sonication technique reported by Zhamu *et al*. The LCA by Arvidsson *et al.* on the sonication production of graphene, based off of the disclosure of Zhamu *et al.* serves to underscore the extremely wide range of approaches and strategies that currently exist in the graphene production industry. Increased understanding of the graphene production process by increasing process transparency and industrial standardization will hopefully result in more test cases, and more detailed and accurate LCA to be carried out as graphene becomes more widespread.

Physical Exfoliation

			Intensity of Inputs				Overall Contribution				
			Cumulative Energy Demand (CED) MJ/unit	100 Year Global Warming Potential (kg CO2 equivalent/unit)	Cumulative Water Use (L/ unit)	Material Input?	Cumulative Energy Demand (CED) MJ	100 Year Global Warming Potential (kg CO2 equivalent)	Cumulative Water Use (L)	Notes	Reference or source
**Ultrasonication**											
Inputs			CED	GWP	CWU		CED	GWP	CWU		
5	kg	Graphite	89.0	4.8	23.7	1	445.0	24.1	118.5		GREET 2020.Net
1000	L	Ethanol								Pristine graphene dispersions even in surfactant assisted solvent systems is extraordinarily low (assumed to be 1 mg/mL here), meaning extremely large amounts of solvent must be used per kg of graphene (1,000 L/kg).	
20	kg	Surfactant								Surfactant identity can vary widely in liquid phase exfoliation of graphene. Due to a lack of useable LCA data on advanced surfactant systems (ie Pluronic F-127), C16–C18 alcohol ethoxylate is utilized as a proxy. A loading of 2 wt% is also assumed here (20 g/L).	
150	kWh	Electricity	7.2	0.45	2.4		1083.2	67.5	361.1	300 W/L @ 0.5 hr	R. Arvidsson, D. Kushnir, B. A. Sandén, and S. Molander, *Environ. Sci. Technol.* , vol. 48, no. 8, pp. 4529–4536, Apr. 2014.
Outputs											
1	kg	Graphene (exfoliated)								Graphene yield from sonication: 20%	H. Zhuo, X. Zhang, L. Wang, Q. Lu, and D. L. Kaplan, *ACS Sustainable Chem. Eng.* , vol. 6, no. 9, pp. 12261–12267, Sep. 2018,
4	kg	Graphite (particles)								Assumed non exfoliated particles remains lower quality graphite and is disposed	
1000	L	Ethanol									
20	kg	Surfactant									

Supplementary Table 3a: Contributions, notes, and sources resulting from the ultrasonication portion of physical exfoliation.

**Centrifugation**											
Inputs											
1	kg	Graphene (suspended)									
1000	L	Ethanol									
0.70	kWh	Electricity	7.2	0.45	2.4		5.1	0.32	1.7	2.11 kW/h/1000L (Perry et al.) for 20 min	Perry, R.H., Green, D.W., Maloney, J.O. (Eds.), 1984. Perry's Chemical Engineers' Handbook, sixth ed. McGraw-Hill, International edition.
Output											
1	kg	Graphene (with mosture)									
975	L	Ethanol (recovered)									
25	L	Ethanol (emitted)	37.0	1.1	27.1	1	925.0	28.0	678.4	The majority of the solvent is recovered after centrifugation (assumed here to be 97%), only 3% of the solvent is calculated here to be lost for every kg of graphite processed.	GREET 2020.Net [Ethanol Denatured Produced in the US (at the Bulk Terminal)
19.4	kg	Surfactant (recovered)									
0.6	kg	Surfactant (lost/emitted)	70.2	2.0		1	42.1	1.2		As the solvent is able to recovered after centrifugation, the majority of surfactant will be recovered after centrifugation. However, significant amounts of surfactant will be bound to the graphene sheets as well as lost during the centrifugation and rinse process, assumed here to be 3%.	D. Schowanek *et al.* , *Int. J. Life Cycle Assess.* , vol. 23, no. 4, pp. 867–886, Apr. 2018

Supplementary Table 3b. Contributions, notes, and sources resulting from the centrifugation portion of physical exfoliation.

**Rinsing/Filtering**											
Inputs			CED	GWP	CWU		CED	GWP	CWU		
1	kg	Graphene (with additional mosture)									
8	L	Ethanol	37.0	1.1	27.1		296.0	9.0	217.1	Assumed solubility of surfactant in ethanol is 125 mg/mL	GREET 2020.Net
--	kWh/MJ	Electricity								Not available; Assumed to be negligible	
Output											
1	kg	Graphene (with additional mosture)									
**Drying**											
Inputs			CED	GWP	CWU		CED	GWP	CWU		
1	kg	Graphene									
0.0025	kWh	Electricity	7.2	0.5	2.4		1.81E-02	1.13E-03	6.02E-03	~0.0025 Wh/g of dried material via vaccum filtration	M. Huttunen et al., Miner. Eng., vol. 100, pp. 144–154, Jan. 2017.
0	MJ	Heat								Assumed latent sonication heat is utilized, thus no additional heat is required	
Output											
1	kg	Graphene									
							CED	GWP	CWU		
					**Total**		**2351.4**	**106.0**	**1258.3**		
					Materials Inputs		1412.14	53.23	796.88		
					Process Energy/Emissions		939	53	461		

Supplementary Table 3c. Contributions, notes, and sources resulting from the rinsing, filtering, and drying portions of physical exfoliation. The grand totals are also shown.

Chemical Exfoliation

					Intensity of Inputs				Contribution				
					Cumulative Energy Demand (CED) MJ/unit	100 Year Global Warming Potential (kg CO2 equivalent /unit)	Cumulative Water Use (L/ unit)	Material Input?	Cumulative Energy Demand (CED) MJ	100 Year Global Warming Potential (kg CO2 equivalent)	Cumulative Water Use (L)	Notes	Reference
					CED	GWP	CWU		CED	GWP	CWU		
**Modified Hummer Process (Oxidation)**													
Inputs	(units)		Density										
1.316	kg	Graphite			89.0	4.8	23.7	1	117	6	31		GREET 2020.Net
7.895	kg	$KMnO_4$			22.8	1.2	62.0	1	180	9	490		R. Randall *et al.* , US Environmental Protection agency, US, 2016.
157.89	L	H2SO4	1.83	kg/L	0.60	0.04	0.32	1	172	12	93		GREET 2020.Net
17.539	L	H3PO4	1.834	kg/L	16.0	1.1	36.7	1	515	34	1180		GREET 2020.Net
11.69993	MJ	Natural Gas			1.1	0.07	0.03		13.4	0.8	0.3	Surphic Acid: Specific Heat 1.34 (kJ/(kg K)); Density@ 40C 1.18107 (g/mL). Heated from 37.5 to 50C for 12 hrs with heat loss of 650 Btu/h (Arvidson 2014)	R. Arvidsson, D. Kushnir, B. A. Sandén, and S. Molander, *Environ. Sci. Technol.* , vol. 48, no. 8, pp. 4529–4536, Apr. 2014.
--	kWh	Electricity			7.2	0.5	2.4					Stirring energy assumed negligible	GREET 2020
Outputs													
2.5	kg	Graphine Oxide											
		$KMnO_4$ (and/or associated products)											
		H_2SO_4 (and/pr associated products)											
		H_3PO_4 (and/or associated products)											

Supplementary Table 4a. Contributions, notes, and sources resulting from the modified Hummer's oxidation portion of chemical exfoliation.

**Quenching, Filtering, Rinsing & Filtering**													
Inputs					CED	GWP	CWU		CED	GWP	CWU		
2.5	kg	Graphine Oxide(s)											
875	L	Water						1			875		GREET 2020.Net
1.316	L	H_2O_2	1.44	kg/L	17	1.06	2.135	1	32	2	4		GREET 2020.Net
Output													
2.5	kg	Graphine Oxide(s)											
		Water											
		H_2O_2 (and/or associated products)											
**Hydrazine Reduction**													
Inputs					CED	GWP	CWU		CED	GWP	CWU		
2.5	kg	Graphine Oxide(s)										Assumption of 40% mass yield from reduction	
582	L	Water						1			582		
1.75	L	Hydrazine	1	kg/L				1	304.2143	18.8354	18.1056	Estimated [below]	
Output													
1	kg	Graphene (rGO)											
		Water											
		Hydrazine											

Supplementary Table 4b. Contributions, notes, and sources resulting from the quenching and hydrazine reduction portion of chemical exfoliation.

**Filtering & Drying**													
Inputs					CED	GWP	CWU		CED	GWP	CWU		
1	kg	Graphene (rGO)											
0.0025	kWh	Electricity			7.221	0.45	2.40765		0.018053	0.001125	0.006019	~0.0025 Wh/g of dried material via vaccum filtration	M. Huttunen et al., Miner. Eng., vol. 100, pp. 144–154, Jan. 2017.
Output													
1	kg	Graphene (rGO)											
									CED	GWP	CWU		
							**Total**		**1333.6**	**83.2**	**3273.2**		
							Materials Inputs		1320.11	82.38	3272.88		
							Process Energy/Emissions		13.5	0.8	0.3		
Hydrazine Production (1 kg)													
65.6	MJ	--NG	(per kg Hydrazine)		1.149318	0.071724	0.026156					Arvidsson et al (2014) estimated process energy required for Hydrazine production.	R. Arvidsson, D. Kushnir, B. A. Sandén, and S. Molander, *Environ. Sci. Technol.* , vol. 48, no. 8, pp. 4529–4536, Apr. 2014.
7.2	MJ	--Electricity	(per kg Hydrazine)		2.005763	0.124721	0.668084						GREET 2020
2	kg	--Ammonia	(per kg Hydrazine)		42	2.58	1.91					Hydrazine is synthesized industrially from reaction of ammonia and chloramine (2:1 stoichiometry), with a 35-fold excess of ammonia, The excess ammonia can be recycled and reused with some loss. Thus, hydrazine material input burdens are assumed 2x ammonia value.	GREET2020.Net

Supplementary Table 4c. Contributions, notes, and sources resulting from the final filtering and drying portion of chemical exfoliation. Totals for the entire process are also shown, as well as calculations of hydrazine contributions.

Flash Joule Heating

			Intensity of Inputs			Overall Contribution				
			Cumulative Energy Demand (CED) MJ/unit	100 Year Global Warming Potential (kg CO2 equivalent/ unit)	Cumulative Water Use (L/ unit)	Material Input?	Cumulative Energy Demand (CED) MJ	100 Year Global Warming Potential (kg CO2 equivalent)	Cumulative Water Use (L)	Notes	Reference
**Hammer Milling**											
Inputs			CED	GWP	CWU		CED	GWP	CWU		
5	kg	Plastic	0	0	0	1	0.0	0.00	0.00	Cut-off approach i.e.	
0.25	kg	Metcoke (MC)	35.0	0.53	0.6	1	8.8	0.13	0.16	Addition of 5 wt%	GREET 2020.Net
0.0945	kWh	Electricity	7.2	0.45	2.4		0.68	0.04	0.23	Proxy: 18 kWh/Mg for Miscanthus biomass hammer milled	Moiceanu, G.; Paraschiv, G.; Voicu, G.; Dinca, M.; Negoita, O.; Chitoiu, M.; Tudor, P. Sustainability 2019, 11 (9), 2477.
Outputs											
5.25	kg	Plastic-MC Mixture									
**LC-FJH and HC-FJH**											
Inputs											
5.25	kg	Plastic-MC Mixture									
30.6	kWh	Electricity (AC)	7.2	0.45	2.4		220.6	13.7	73.6	5.8 Wh/g of plastic waste mixture	Algozeeb, W. A.; Savas, P. E.; Luong, D. X.; Chen, W.; Kittrell, C.; Bhat, M.; Shahsavari, R.; Tour, J. M. ACS Nano 2020, 14 (11), 15595–15604. Electricity contributions source: GREET 2020
4.4	kWh	Electricity (DC)	7.2	0.45	2.4		31.8	2.0	10.6	3.6 Wh/g per AC flash graphene (1.1 kg AC-FJH : 1 kg graphene); assume 90% inverter efficiency	
Output											
1	kg	Graphene									
0.45	kg	CO2						0.45		Assumed: Process offgas composed of 0.12 kg of CO2 and 0.12 kg of CH4 (which is abated by combustiong, yielding 0.33 kg of additional CO2)	Algozeeb, W. A.; Savas, P. E.; Luong, D. X.; Chen, W.; Kittrell, C.; Bhat, M.; Shahsavari, R.; Tour, J. M. ACS Nano 2020, 14 (11), 15595–15604.

Supplementary Table 5a. Contributions, notes, and sources resulting from the sample preparation and FJH portions of the FJH synthesis process.

			Cumulative Energy Demand (CED) MJ	100 Year Global Warming Potential (kg CO2 equivalent)	Cumulative Water Use (L)
		**Total**	**262**	**16**	**85**
Materials Inputs			8.75	0.13	0.16
Process Energy/Emissions			253	16	84

Supplementary Table 5b. A table totaling the contributions resulting from the FJH synthesis process.

Summary of Results (per 1 kg graphene powder)

	Cumulative Energy Demand (CED) MJ			Global Warming Potential (kg CO2 equivalent)			Cumulative Water Use (L)		
	Materials	Process	**Total**	Materials	Process	**Total**	Materials	Process	**Total**
FJH	9	253	**262**	0	16	**16**	0	84	**85**
Physical Exfoliation	1412	939	**2351**	53	53	**106**	797	461	**1258**
Chemical Exfoliation	1320	14	**1334**	82	1	**83**	3272	1	**3273**

Supplementary Table 6. Summarizing the findings of the preliminary LCA.

SI references:

1) S Ghasemi, *et. al.* Flexible polyurethane foams reinforced with organic and inorganic nanofillers. *J. Appl. Polym. Sci.,* **138**, 49983 (2021).
2) Bote, S.D., Kiziltas, A., Scheper, I., Mielewski, D., Narayan, R. Biobased flexible polyurethane foams manufactured from lactide-based polyester-ether polyols for automotive applications *J. Appl. Polym. Sci.* **138**, 50690 (2021).
3) Serrano-Luján, L. *et. al.* Environmental impact of the production of graphene oxide and reduced graphene oxide. *SN Appl. Sci.* **1**, 179 (2019)
4) Arvidsson, R.; Kushnir, D.; Sandén, B. A.; Molander, S. Prospective life cycle assessment of graphene production by ultrasonication and chemical reduction. *Environ. Sci. Technol.* **48**, 4529–4536 (2014)
5) Pizza, A.; Metz, R.; Hassanzadeh, M.; Bantignies, J.-L. Life cycle assessment of nanocomposites made of thermally conductive graphite nanoplatelets. *Int. J. Life Cycle Assess.* **19**, 1226–1237 (2014)
6) Cossutta, M.; McKechnie, J.; Pickering, S. J. A Comparative LCA of different graphene production routes. *Green Chem.* **19**, 5874–5884 (2017)
7) Hernandez, Y. *et. al.* High-yield production of graphene by liquid-phase exfoliation of graphite. *Nat. Nanotechnol.* **3**, 563–568 (2008)
8) Zhamu, A.; Jang, B. Z. Mass Production of Pristine Nano Graphene Materials, US Patent 8,226,801 B2; Nanotek Instruments Inc.
