# Supplementary material for: Upcycling end-of-life vehicle waste plastic into flash graphene
Source: Commun Eng. 2022 May 26;1:3. doi: 10.1038/s44172-022-00006-7 (PMC10955767; doi:10.1038/s44172-022-00006-7)
Supplement: Supplementary file 4 — Description of Additional Supplementary Files [file 44172_2022_6_MOESM4_ESM.pdf]

Description of Additional Supplementary Files

File Name: Supplementary Data 1

Description: Spreadsheet showing detailed calculations and references used in the perspective LCA.

File Name: Supplementary Data 2

Description: Source data for figures in the body of the manuscript
